# Extraction of niclosamide from commercial approved tablets into aqueous buffered solution creates potentially approvable oral and nasal sprays against COVID-19 and other respiratory infections

**DOI:** 10.1186/s41120-023-00072-x

**Published:** 2023-04-14

**Authors:** David Needham

**Affiliations:** 1 Department of Mechanical Engineering and Material Science, Duke University, Durham, NC 27708 USA; 2 School of Pharmacy, University of Nottingham, Nottingham, NG7 2RD UK

**Keywords:** Niclosamide, Dissolution, Yomesan, Luxiaoliunpian, Niclosig, pH buffer, Universal nasal-throat-spray, COVID-19, Respiratory viral infections

## Abstract

**Motivation:**

The low solubility, weak acid drug, niclosamide is a host cell modulator with broad-spectrum anti-viral cell-activity against many viruses, including stopping the SARS-CoV-2 virus from infecting cells in cell culture. As a result, a simple universal nasal spray preventative was proposed and investigated in earlier work regarding the dissolution of niclosamide into simple buffers. However, starting with pharmaceutical grade, niclosamide represents a new 505(b)(2) application. The motivation for this second paper in the series was therefore to explore if and to what extent niclosamide could be extracted from commercially available and regulatory-approved niclosamide oral tablets that could serve as a preventative nasal spray and an early treatment oral/throat spray, with possibly more expeditious testing and regulatory approval.

**Experimental:**

Measurements of supernatant niclosamide concentrations were made by calibrated UV-Vis for the dissolution of niclosamide from commercially available Yomesan crushed into a powder for dissolution into Tris Buffer (TB) solutions. Parameters tested were as follows: time (0–2 days), concentration (300 µM to -1 mM), pH (7.41 to 9.35), and anhydrous/hydrated state. Optical microscopy was used to view the morphologies of the initial crushed powder, and the dissolving and equilibrating undissolved excess particles to detect morphologic changes that might occur.

**Results:**

*Concentration dependence*: Niclosamide was readily extracted from powdered Yomesan at pH 9.34 TB at starting Yomesan niclosamide equivalents concentrations of 300 µM, 600 µM, and 1 mM. Peak dissolved niclosamide supernatant concentrations of 264 µM, 216 µM, and 172 µM were achieved in 1 h, 1 h, and 3 h respectively. These peaks though were followed by a reduction in supernatant concentration to an average of 112.3 µM ± 28.4 µM after overnight stir on day 2. *pH dependence*: For nominal pHs of 7.41, 8.35, 8.85, and 9.35, peak niclosamide concentrations were 4 µM, 22.4 µM, 96.2 µM, and 215.8 µM, respectively. Similarly, the day 2 values all reduced to 3 µM, 12.9 µM, 35.1 µM, and 112.3 µM. A heat-treatment to 200 °C dehydrated the niclosamide and showed a high 3 h concentration (262 µM) and the least day-2 reduction (to 229 µM). This indicated that the presence, or formation during exposure to buffer, of lower solubility polymorphs was responsible for the reductions in total solubilities. These morphologic changes were confirmed by optical microscopy that showed initially featureless particulate-aggregates of niclosamide could grow multiple needle-shaped crystals and form needle masses, especially in the presence of Tris-buffered sodium chloride, where new red needles were rapidly made. *Scale up*: A scaled-up 1 L solution of niclosamide was made achieving 165 µM supernatant niclosamide in 3 h by dissolution of just one fifth (100 mg niclosamide) of a Yomesan tablet.

**Conclusion:**

These comprehensive results provide a guide as to how to utilize commercially available and approved tablets of niclosamide to generate aqueous niclosamide solutions from a simple dissolution protocol. As shown here, just one 4-tablet pack of Yomesan could readily make 165 L of a 20 µM niclosamide solution giving 16,500 10 mL bottles. One million bottles, from just 60 packs of Yomesan, would provide 100 million single spray doses for distribution to mitigate a host of respiratory infections as a universal preventative-nasal and early treatment oral/throat sprays throughout the world.

**Graphical Abstract:**

pH dependence of niclosamide extraction from crushed Yomesan tablet material into Tris buffer (yellow-green in vial) and Tris-buffered saline solution (orange-red in vial). Initial anhydrous dissolution concentration is reduced by overnight stirring to likely monohydrate niclosamide; and is even lower if in TBSS forming new niclosamide sodium needle crystals grown from the original particles.

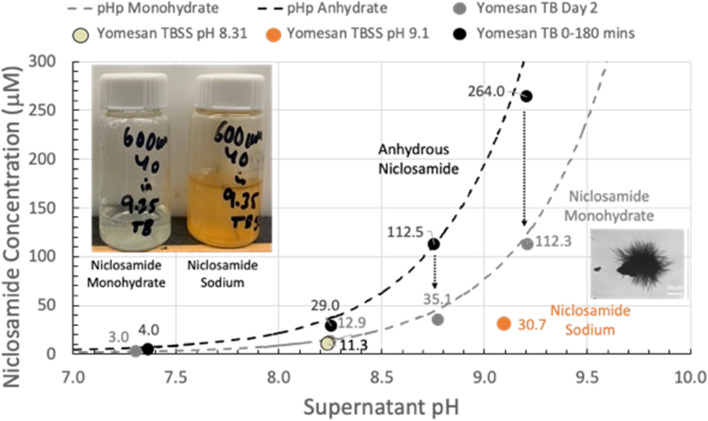

**Supplementary Information:**

The online version contains supplementary material available at 10.1186/s41120-023-00072-x.

## Introduction

This is the second paper in a series of studies starting in 2021–2022 (Needham 2021, 2022) that report on the potential application of a simple aqueous buffered solution of niclosamide as a potential preventative nasal spray and early treatment throat spray for COVID-19 and other respiratory virus infections. While ostensibly simple in concept, a considerable amount of data is required to comprehensively characterize the solubility, dissolution, morphology, polymorphic states, precipitation, and interactions with common mucin-binding cellulosic polymers that would go into an effective formulation. This current paper departs from using pure niclosamide and instead quantifies the dissolution of niclosamide from commercially available, powdered, Yomesan tablet as a function of concentration, pH, and suspected polymorphs and includes a scale up to the 1 L scale.

*Yomesan* (Bayer, Germany) is approved for human use and was chosen because it is the most widely commercially available niclosamide tablet. Two other approved tablets are also available—the human-generic *Niclosig* (HAB Pharmaceuticals and Research Ltd., India) and the livestock-generic *Luxiaoliunpian* (Hanzhong Tianyuan Pharmaceuticals, China). These were also investigated for comparison and gave similar results to those reported for Yomesan. However, because their excipient compositions were not provided by the manufacturers, and the already considerable length of the paper, the full data is not reported here (interested parties please contact the author for details).

The data presented is designed to guide the making of a simple buffered solution of niclosamide using already approved tablet materials. These solutions, when tested, could have application in a range of respiratory viral infections including COVID-19, influenza, and RSV.

### Scientific motivation

#### Preformulation drug characterization

Niclosamide is a notoriously low solubility drug in aqueous media. It can be present in one of several anhydrous and hydrated polymorphs, each having different water solubilities, thereby affecting formulation and clinical utility (de Villiers et al. 2004). Pure niclosamide has an intrinsic solubility of ~ 1–2 µM for its most stable monohydrate H_B _polymorph (Needham 2022; van Tonder et al. 2004). Other intranasal formulations under current clinical testing (Backer et al. 2021; Sommer et al. 2021; Weiss et al. 2021) have sought to increase the amount of niclosamide in solution by resorting to niclosamide ethanolamine (NE) that is slightly more soluble, but is generally more toxic, as a skin, eye, and respiratory irritant (AKSci 2022; Cayman-Chemicals 2019; DHSS 2002). As detailed briefly below, while data by others (Gassen et al. 2020; Jeon et al. 2020; Ko 2020b; Wu et al. 2004; Braga et al. 2021; Brunaugh et al. 2021; Cairns et al. 2022) shows that activity of niclosamide against SARS-CoV-2 in in vero6 and other cells is on the order of 1 - 2 uM, our new (unpublished) data in human nasal (HNE) and bronchial (HBE) epithelial cells shows that niclosamide starts to become lethal at 100 micromolar (for a 24hr exposure). This is therefore much less than in the Union Therapeutics formulation (Sommer et al. 2021), where NE is solubilized in cyclodextrin and dosed at 25mM. Apart from the hazard warnings about niclosamide ethanolamine, one other fear is that niclosamide, encapsulated or artificially solubilized in any aqueous media, will eventually equilibrate to its most stable monohydrate and lowest solubility form upon storage or transport. Actual aqueous bioavailability is set by the thermodynamic solubility of the niclosamide in equilibrium with this lowest soluble and most stable form, and so solubilization could be somewhat moot, i.e., it is what is in solution that counts, hence the need to characterize the drug first before committing to any “solubilizing formulation.”

Recognizing that niclosamide is a weak acid with a pKa ~ 7.12, the data in the earlier paper (Needham 2022) showed that excess pure, probably anhydrous (AK Sci), niclosamide could readily dissolve and equilibrate in aqueous buffered solution. Solubilities ranged from an intrinsic solubility of 2.53 µM at pH 3.66 to 30 µM a nasally safe pH 8.3, just by slightly increasing the alkalinity. The concentration can be raised to 200 µM at pH 9.0, and 300 µM at pH 9.2 where the concentration vs. pH curve becomes quite steep. At an orally safe pH 9.63, where the unprotonated salt dominates, the amount of niclosamide in solution can be increased to 703.6 µM—all in simple Tris buffer, with no other added agents. Thus, if slightly alkaline pH can be tolerated, the amount of niclosamide in simple aqueous solution could be almost 1000 times its IC_100_that completely inhibits SARS-CoV-2 viral replication as measured previously in vero6 and other cells.

Interestingly, there was some dependence of solubility on the polymorphic nature of the niclosamide obtained from certain suppliers (AK Sci and Sigma), and a pH dependence of the solubility of all polymorphs and solvates was shown (for the first time) to exist across the whole pH range (Needham 2022).

#### Scientific questions for the tablets

The questions addressed here, then, are, “Could niclosamide be simply extracted from commercial tablets into pH buffer?” (i.e., no organic solvents). If so, this might represent a more expedited path to regulatory approval (see below). For its original anti-helminthic application, Yomesan tablets are taken orally in 2 g quantities. In contrast, a nasal or throat spray would simply dissolve the niclosamide in an excess of aqueous buffer and only deliver a few micrograms of soluble niclosamide per dose. Also, as a point of pharmaceutics, “What if the niclosamide used in various manufactured tablets was also polymorphic? “How would this affect the drug extraction and aqueous solubility?” i.e., “What source of niclosamide is used in these tablets and are some anhydrous and some more hydrated crystalline forms, or even a mixture?” The reason this is important is that the known interconversion between the anhydrous and monohydrate polymorphs (de Villiers et al. 2004; van Tonder et al. 2004) could affect niclosamide’s solubility and hence the amount of niclosamide that could be extracted, especially in a time-dependent manner.

### Clinical motivation

As yet, no niclosamide nasal or throat spray is clinically available. Once characterized, as done here, a simple procedure could be established for such extraction into sterile buffer, shared, and widely disseminated, especially to compounding pharmacies in parts of the world at most risk of respiratory infection and perhaps lack of available vaccines and other medicines.

#### Why niclosamide?

As reviewed earlier (Gassen et al. 2020; Jeon et al. 2020; Jurgeit et al. 2012; Ko et al. 2021a, b; Wu et al. 2004; Xu et al. 2020), the reappropriation of niclosamide is well-motivated by data in vero6 and calu-3 cell culture showing that it has activity against SARS-CoV-2 (Gassen et al. 2020; Jeon et al. 2020; Ko 2020b; Wu et al. 2004; Braga et al. 2021; Brunaugh et al. 2021; Cairns et al. 2022) and broad spectrum activity against other respiratory viral infections (Xu et al. 2020).

Many current anti-virals either attempt to disrupt the synthesis and assembly of viral proteins (viral proteases, RNA-dependent RNA-polymerase, virus helicases, viral spike and structural proteins) or target host proteins and mechanisms required by the viral replication cycle (including boosting interferon response, ACE2 receptors, cell surface and endosomal proteases, and clathrin-mediated endocytosis) (Laise et al. 2020). Niclosamide offers a different and potentially very effective way to combat viral infection because it enters host cell membranes as a lipophilic anion where it acts as a proton shut, dissipating pH gradients across a range of host cellular-organelle membranes, including mitochondria, endosomes, and lysosomes, and even the Golgi. These mechanisms also apply beyond viral infection. In an excellent and comprehensive review, Chen et al. (2018), and recently updated by Wang et al. (2022) for pharmacological activities and therapeutic prospects, report that niclosamide, tested mainly in cells and a few preclinical animal cancer models, has efficacy that includes “cancer, bacterial infection, metabolic diseases such as type II diabetes, NASH and NAFLD, artery constriction, endometriosis, neuropathic pain, rheumatoid arthritis, sclerodermatous graft-versus-host disease, and systemic sclerosis”. There are also reports of activity in Parkinson’s (Barini et al. 2018) and COPD (Cabrita et al. 2019; Miner et al. 2019). Niclosamide is a career builder should anyone want to study it in detail.

#### Estimated niclosamide concentrations for the sprays

The estimated niclosamide concentrations for the sprays are guided by our recent studies in the more respiratory-relevant human nasal and bronchial epithelial cells. These as yet unpublished data show that niclosamide can become lethal (by Lactate Dehydrogenase Assay) at concentrations approaching ~ 100 µM (for a 24-h exposure). We therefore sought to keep the bioavailable niclosamide solution concentrations below this level. Target concentrations for a preventative nasal spray would therefore be ~ 20–30 µM at a nasally safe pH 8.3 and could be as high as 300 µM at pH 9.3 as an early treatment throat spray for a sore throat, which is becoming the dominant COVID symptom (Carolyn 2022). At these levels, the amounts of niclosamide in the sprays are tiny compared to the 2 g of niclosamide in the approved oral dosing of the tablets. To be accurate, we need to consider the *bioavailable soluble fraction* of 2 g. Using a solubility of 1 µM in 30 mL of water or saliva, this is only ~ 9.8 µg. Comparatively, 100 µL of a 300 µM sprayed oral dose of niclosamide solution is also 9.8 µg and so is on the same order as the approved and safe oral tablet dose. An intranasal 20 μm dose is only 0.65 μg (see Additional file 1: Supplementary Information, S8 on Comparative dosing).

#### Expedited regulatory approval?

If already pharmaceutical grade niclosamide could be simply extracted from crushed approved tablets that are normally taken by oral ingestion, the need for a “new” formulation and extensive regulatory approval could perhaps be expedited. That is, an early treatment throat spray, and even nasal spray when tested, could be obtained by following Bayer’s recommended current dosing regimen of dispersing 4 × 500 mg tablets in a small amount of tap water (Bayer 2008), but instead, use excess aqueous buffer to give a final filtered niclosamide solution. As calculated later, one packet of Yomesan could generate tens of liters of a 20 µM niclosamide solution that could be filled and finished into 10 mL conventional spray bottles and be readily sprayed orally, and also nasally.

With infection-acquired immunity failing, new vaccines and boosters needed to keep up immunity, a decrease in compliance with vaccines and boosters across the population, the recent failure of the nasal spray Oxford/AstraZeneca Covid vaccine (Madhavan et al. 2022; Sample 2022), and the emergence of a more infectious and antibody-escaping BQ.1.1 subvariant (Axe 2022), there is even more urgency to develop, test, and make available simple and effective preventatives and early treatment options for infection and spread. A simple niclosamide solution has the potential to be the basis for that.

## Experimental

### Materials

Tablet material was *Yomesan* (approved human pharmaceutical) from Bayer. Others were also tested but their full results are not reported here; they were the generic *Niclosig* (HAB Pharmaceuticals), as well as a live-stock generic *Luxiaoliuanpian* (abb. Lux) from Hanzhong Tianyuan Pharmaceuticals, China.

*Yomesan *(Bayer, Leverkusen, Germany): Although contents for Yomesan were listed in their package insert (Bayer 2008), their respective concentrations were not available. Yomesan consists of 500 mg niclosamide; maize starch (C_27_H_48_O_20_, 692.7 g/mol, solubility 3.22%, 46.5 mM); talcum—magnesium silicate (H_2_Mg_3_O_12_Si_4_, 379.27 g/mol, considered insoluble in water); sodium lauryl sulphate; povidone (polyvinylpyrrolidone); Vanillin (C_8_H_8_O_3_); magnesium stearate (Mg (C_18_H_35_O_2_)_2_); and saccharin sodium (C_7_H_4_NNaO_3_S).

Water was deionized and filtered through 0.22 μm filters (VWR acrylic filters). TRIZMA buffer was from Supelco and comprised of the following: Trizma Base, ≥ 99.8%, reagent grade tris(hydroxymethyl)-aminomethane (HOCH_2_)_3_CNH_2_, Mol Wt 121.14 g/mol, white crystalline powder; Trizma HCl, ≥ 99%, reagent-grade (tris[hydroxymethyl] aminomethane hydrochloride), (HOCH_2_)_3_CNH_2_•HCI Mol Wt 157.60 g/mol, white crystalline powder. Tris buffer was used mostly at 20 mM buffer concentration. pH buffers were made using the TRIZMA HCl and TRIZMA base buffer system according to recipes at AAT Bioquest (AATbio.com 2021). Vanillin was from MedChem Express (MCE), Cat:HY90098-CS-W020052. Ethanol KOPTEC 200% proof was from VWR. Corn starch was from Lab Alley, TX, and USP talc from Medisca, both kindly provided by Vincent Gaver, PharmD, BCPS, Duke Investigational Drug Services.

#### Niclosamide equivalents (mg niclosamide/mg tablet)

Because of the presence of excipients in addition to the 500 mg of niclosamide, total niclosamide concentrations added to the buffers were calculated based on a *niclosamide-equivalent* per tablet material, i.e., mg niclosamide/mg of tablet powder. For Yomesan, a 500 mg niclosamide tablet weighed 638 mg, and therefore, 138 mg of the tablet was from excipients, including the typical talc and corn starch (as binder and disintegrants), and other excipients. The tablet material was therefore 0.784 mg niclosamide/g of tablet. Under the microscope, these excipients were confirmed to be corn starch and talc and were compared to test images of pure USP corn starch and USP talc.

### Research design

As detailed in the previous publication (Needham 2022), equilibrium and dissolution rates for pure niclosamide purchased from commercial suppliers, AK Sci. and Sigma, were established over a range of pHs (3.5–9.5) and under optimized stirring conditions. As the pH of the buffer was increased, the total solubility of niclosamide concomitantly increased from an intrinsic solubility at pH 3.5 for the protonated acid of 2.53 to 703 µM at pH 9.63 where, again, the unprotonated, negatively charged, more-soluble salt dominates. Such solubility limit data followed precipitation pH and Henderson-Hasselbalch theories. In the current work, it was hypothesized that if niclosamide could readily dissolve in pH buffers then it could be readily pH-extracted from crushed commercial tablets (i.e., in the absence of organic solvents). The Yomesan tablet is the most common and widely available niclosamide product^1^. The dissolution, and hence extraction, of niclosamide from the crushed tablet material was therefore investigated for the commercially available and approved niclosamide tablet, *Yomesan*. Others (Niclosig and Luxiaoliuanpian) were tested and relevant data will be mentioned in passing.

The overall goal was to determine if and to what extent niclosamide in the tablets could be extracted into pH buffer by simply grinding up the tablet into a relatively fine powder using a common pill-crusher, and adding excess of this powdered material, including its excipients, into 20 mL of Tris-buffered solutions. The research design for this series of studies was relatively straightforward and included two main specific aims (SA) with additional sub-aims in Tris-buffered saline solution (TBSS) and scale up to the 1 L scale.

#### SA1. Concentration dependence

##### To measure the extraction of niclosamide from Yomesan tablets in Tris-buffered solution at nominal pH 9.35 over a range of niclosamide-equivalent concentrations (300 µM, 600 µM, and 1 mM)

Experiments were designed to demonstrate that niclosamide can be obtained in supernatant solution at potentially efficacious oral and nasal spray concentrations, i.e., ~ 20 µM at pH 8.3 for nasal, and up to 300 µM at pH 9.3 for oral administration. Excess amounts of Yomesan powder were added to the buffers at niclosamide-equivalent concentrations of 300 µM, 600 µM, and 1 mM. These concentrations were based on the pH-dependent total solubility limits (i.e., of protonated niclosamide acid plus niclosamide deprotonated salt) measured earlier (Needham 2022). These earlier measurements on pure niclosamide were actually done on, what now appears to be, the anhydrous niclosamide polymorph obtained from AK Sci (unpublished X-ray diffraction data obtained by Akash Singh, in the Mitzi Lab, Duke University). This AK Sci niclosamide proved to be the most soluble polymorph compared to the monohydrate, H_A_ and H_B_ polymorphs.

In the current experiments, after addition of the dry powdered Yomesan to 20 mL of buffer solution, small aliquots (0.7 to 0.8 mL) were taken from the stirred suspension at regular time intervals, filtered through a 0.22 µM filter and full UV-Vis spectra obtained using a niclosamide-calibrated UV-Vis spectrometer. The peak at 333 nm was used to provide the niclosamide concentration in the filtered supernatant.

Preliminary experiments showed the presence of a UV-Vis absorbing excipient “impurity” that was likely vanillin, detected in Yomesan (but not in the other two products). As described in the “Methods” section, this was accounted for by subtracting the 30 s spectrum from the subsequent measurements (see also Additional file 1: Supplementary Information, S2. Vanillin).

Optical microscopy (Nikon Diaphot equipped with 10× and 40× bright field objectives) was used to provide additional observations of the morphological nature of the original powder and suspended excess material as it equilibrated with niclosamide in solution. The thinking here was that if any morphological changes occurred compared to the original polymorphic nature of the niclosamide (anhydrous, H_A_, H_B,_ or mixture thereof), microscopic observations could detect such changes and motivate future analytical studies.

##### Heating to create the anhydrous form

As an initial check on the possibility that Yomesan contained one or more polymorphs, following van Tonder et al. (2004) and de Villers et al. (2004), a heating experiment was carried out to detect water of crystallization by simple condensation on a glass coverslip during the heating ramp. Continued heating to 200°C for 15 min provided dehydrated anhydrous Yomesan-niclosamide for subsequent dissolution testing. Future tests and analytical characterizations could therefore involve thermo-gravimetric (TG) and differential scanning calorimetric (DSC) measurements to quantify these waters of hydration in the Yomesan-niclosamide and XRD and Raman studies to confirm their polymorphism.

#### SA2. pH dependence

##### To measure the extraction of niclosamide from Yomesan into (nominal) pH 7.41, 8.35, 8.85, and 9.35 Tris buffer

Having already discovered and demonstrated that the total solubility of pure niclosamide has a pH dependence, this second series of experiments was designed to explore the pH dependence of the dissolution of niclosamide from excess amounts of crushed Yomesan powder. Dissolution was carried out in Tris-buffered aqueous solutions at four nominal pHs, 7.40, 8.35, 8.85, and 9.35.

Excess crushed tablet material was again added to the buffer in amounts that would be over and above its expected pH-dependent total solubility measured earlier (Needham 2022), i.e., 5 µM at pH 7.0, 41 µM at pH 8.3, 137 µM at pH 8.85, and 380 µM at pH 9.3. Thus, Yomesan niclosamide-equivalent concentrations of 300 µM for pH 7.4 and 600 µM for pHs 8.35, 8.85, and 9.35 provided enough excess powder in suspension for niclosamide extraction into the buffered solutions.

Optical microscopy was again used to provide additional observations of the morphological nature of the original powder and suspended excess material as it equilibrated over time (out to day 2 and sometimes beyond) with the excess undissolved niclosamide in solution.

##### Extraction into Tris-buffered saline solution (TBSS)

Some interesting preliminary data were obtained when Yomesan powder was dissolved in Tris-buffered saline solution (TBSS). The original thinking was to use such TBSS in order to provide a niclosamide solution of higher osmolality, so that it approached or matched the nasal osmolality of ~ 290 mOsm. However, equilibrating in TBSS gave a rapid conversion of the undissolved excess powder material to a red-colored needle-like morphology. This new polymorph had a lower solubility than the monohydrates and so lowered the available niclosamide in supernatant solution even further. This data is included to again motivate future studies on what seems to be a new observation, that excess niclosamide in the presence of saline can rapidly convert to what appears to be a niclosamide sodium salt or it could be a co-crystal as observed by Grifasi et al. (2015) for kneaded dry samples.

##### To scale-up dissolution to a 1 L scale

Finally, to demonstrate the effectiveness of the procedure and start to develop a scaled-up protocol, dissolution of fresh-crushed Yomesan powder was carried out at the 1 L scale. One fifth of a Yomesan tablet is 125 mg and so provides 100 mg of niclosamide-equivalent which, in 1 L of buffer, is ~ 300 µM total concentration of added niclosamide. This concentration was chosen because, as later results showed, even though it did not give the highest supernatant niclosamide concentration, the 300 µM total added niclosamide-equivalent gave the best extraction yield of almost 60% (see also Additional file 1: Supplementary Information, S4. Fraction of Niclosamide Extracted from Yomesan Powder).

### Methods

#### Crushing tablet materials

**Fig. 1:**
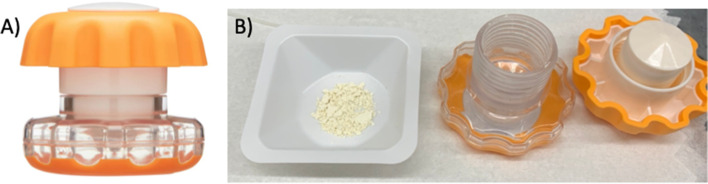
Preparation of crushed Yomesan tablet for dissolution into Buffers. **A** Equate, Easy-Grip Pull Crusher, used to crush whole Yomesan (Niclosig and Lux) tablets. **B** 637.7 mg of a 500 mg niclosamide crushed Yomesan tablet (i.e., 0.784 mg niclosamide per 1 mg of tablet)

Tablet dissolution experiments started with whole tablets of Yomesan that were crushed into a fine powder using a common pill crusher (Equate Easy Grip Pill Crusher), shown in Fig. 1A. As shown in Fig. 1B, the 500 mg niclosamide-Yomesan tablet yielded 637.7 mg of crushed material due to excipients, i.e., 0.784 mg of niclosamide/1 mg of tablet.

#### Initial weighing

Thus, to obtain excess niclosamide concentrations for addition to the buffered solutions, it was necessary to take into account the presence of excipients when weighing out the powder as mg of niclosamide per mg of tablet. For example, to provide ~ 1 mM niclosamide total in 20 mL of buffered solution, tablet powder was weighed as 8.33 mg Yomesan (12.95 mg Niclosig, 8.65 mg Lux). A convenient way to weigh such small amounts of dry crushed powder was into clean Eppendorf tubes, ready for addition to the stirred buffer at time zero.

#### The series of timed dissolution studies (0–180 min and day 2): concentration, pH, salt, and scale-up

Timed dissolution studies were carried out in 20 mL of Tris buffer in a 20 mL scintillation vial, stirred gently on a magnetic stir plate at a stir bar speed of 300 rpm. As above, two main specific aims were carried out: *concentration-dependent dissolution* for 300 µM, 600 µM, and 1 mM Yomesan-niclosamide at pH 9.35 and *pH-dependent dissolution-extraction* in Tris buffer at nominal pHs of 7.41, 8.35, 8.85, and 9.35. Starting total concentrations for this pH dependence study were 300 µM total equivalent of niclosamide at pH 7.4 and 600 µM for the three higher nominal pHs 8.35, 8.35, and 9.35. The final pH of the equilibrated niclosamide suspension often decreased slightly and was measured during dissolution and after equilibration on day 2.

Similar studies were also done in 20 mM Tris buffer made in saline solution (100 mM NaCl) (TBSS). A 1 L scale up was also performed at 125 mg/L Yomesan powder, giving a 100 mg/L equivalent niclosamide concentration. The solution was recovered by vacuum filtering (Thermo Scientific™ Nalgene™ Rapid-Flow™ Sterile Disposable Filter Units with PES Membranes).

##### Sampling

Measurements were made by sampling the stirred suspension at regular intervals on day 1, from 30 s to 180 min, and then again on day 2, ~ 24 h after addition of material and continual overnight stirring. All dissolution experiments were done at room temperature 23 °C. Twenty milliliters of buffer was enough to allow for sampling during the dissolution process. At each time point, just less than 1 mL of supernatant was taken into a BD 1 mL syringe at the prescribed times (30 s, 1 min, 2.5 min, 5 min, 10 min, 15 min, 20 min, 25 min, 30 min, 40 min, 50 min, 60 min, 75 min, 90 min, 120 min, 150 min, 180 min, and ~ 24 h). As judged by a lab stop-clock, samples were withdrawn into the 1 mL syringe through a blunt needle, timing the 2 s withdrawal at 59 s to 01 s around the time point. Samples were filtered through small volume 0.22 µm Cytiva Whatman™ Anotop™ 10 mm Syringe Filters (Cytiva 68091022) to remove excess suspended particulate tablet materials. The ~ 0.5 mL of filtered sample was always placed in the same low-volume plastic cuvette that had been used as the blank with buffer and was cleaned between measurements.

For some of the early timed dissolution studies (in Tris-buffered saline), a series of plastic cuvettes were lined up and labeled for their respective sample times. It was however found that each plastic cuvette actually produced a slightly different blank UV-Vis absorbance spectrum that, while not significant for the higher concentrations gave variations in absorbances that were on the same order as those for the lower 2–4 µM concentrations, for example, early in the dissolution process (see Additional file 1: Supplementary Information, S7 Comparative UV-Vis Spectra) and at pH 7.4 (see Supplemental Information S7.2 pH Dependence, pH 7.41 Fig. S9A).

While this was allowed for by a cuvette correction, for most of the experiments, the technique was optimized, and the same cuvette was used for all measurements, again cleaning between each measurement.

##### UV-Vis measurements

Niclosamide concentrations of the sample supernatants were determined by UV/Vis analysis (Mettler Toledo UV5nano, LabX software) via calibration of a series of niclosamide solutions in pH 9.35 Tris buffer (10–300 µM). All measurements were done at room temperature 23 °C. This high pH was used because the range for niclosamide solubility could be increased to 300 µM. It should be noted though that, at these higher concentrations of 300 µM, the relative absorbances were ~ 2.5 absorbance units (AU). As such, the UV absorbance through the 1 cm pathlength cuvette reached the limit of detection of the photomultiplier tube. As expected from Beer-Lambert, spectra became noisy from the low intensity of the signal reaching the detector (see Additional file 1: Supplementary Information, S7. Comparative UV-Vis Spectra, Fig. S8). Samples were therefore diluted 2× by adding 0.5 mL of filtered control buffer to 0.5 mL of the filtered sample in the cuvette to give readings in the acceptable range of the instrument.

Full UV-Vis spectra (190–1100 nm) were captured (at 0.2 nm intervals) using the Mettler Toledo LabX software and processed in Microsoft Excel (see Additional file 1: Supplementary Information, S7. Comparative UV-Vis Spectra, S7.1 Concentration-dependence and S7.2 pH-Dependence). The wavelength range was reduced to 290–700 nm for easier handling that contained the main peaks of interest for niclosamide at 333 and 377 nm. This spread-sheet manipulation allowed several corrections and subtractions to be made, including a 3-measurement average and standard deviation, corrections due to individual cuvette absorbance (as used in preliminary experiments), baseline corrections (to 500 nm that was outside the absorbance range), and subtractions of the “impurity” vanillin excipient from the Yomesan samples (see Supplemental Information, S2. Vanillin).

Also, in order to measure the low 2–4 µM solubilities of the niclosamide supernatant at pH 7.4, the ultimate sensitivities and limits of the UV-Vis technique were carefully established. A single quartz cuvette was used (in the same orientation each time) to measure three buffer samples and deionized water, cleaning between each of three-averaged measurements. The reported photometric resolution of the UV5 Nano is ± 0.005 AU for the 1 cm cuvette path length; this was checked and confirmed (see Additional file 1: Supplementary Information, S1. Instrument Resolution (Photometric accuracy) Fig. S1).

##### Excipient “impurity” corrections

Preliminary studies showed that Yomesan contained an excipient that absorbed in the same 300–400 nm range as niclosamide. Experimentation showed that this excipient was likely the taste-masking compound vanillin (see Additional file 1: Supplementary Information, S2. Vanillin). A concentration calibration was therefore carried out to establish the spectral identity and range as a function of vanillin concentration in Tris buffer. It was actually found that its spectrum at pH 7 comprised two peaks, compared to the single peak at pH 8.35 and 9.35. This is likely associated with vanillin being a weak acid. (This vanillin “impurity” was not present in the Niclosig and Lux generics.)

Other excipients included corn starch and talc. Starch paste, at a concentration of 5–20% finds applicability as a disintegrant in a number of tablet formulations (Manek et al. 2012). Thus, samples of both talc and cornstarch were obtained, and spectra of their saturated solutions were determined. Because of their low water solubility, absorbances were very low on the same order as the instrument resolution (± 0.005 AU) and did not significantly impact the UV-Vis spectra of niclosamide (see Additional file 1: Supplementary Information, S3 Talc and Corn Starch and Fig. S4). In any event, all this was accounted for by early sampling at the 30 s time point providing a subtractable initial spectrum.

##### pH measurements

Solution and suspension pHs were measured using a Mettler SevenEasy™ pH Meter S20, calibrated prior to measurements using standard (VWR) buffers of pH 4, 7, and 10.

### Additional characterization studies

#### Bright field optical microscopy

Bright field optical microscopy was used for further characterization of the nature of the excess undissolved and equilibrated material. During equilibration, the excess undissolved powder often underwent a morphologic change signifying a conversion to, or crystal growth of, a new polymorph. The reason this is important is that that any new polymorph could have a different (usually lower) solubility and so influence the equilibrating amount of niclosamide that was obtained in solution. Optical microscopy was therefore used to observe the initial nature of the dry crushed powders, the powders suspended in water, and the excess particles suspended in the dissolution buffers during and after dissolution equilibrium. These real-time observations, that are not usually done in the literature (which is mostly SEM), gave new insights into the morphological changes that occurred especially for Yomesan-niclosamide during the dissolution of the excess powder. Using a home-built glass slide + cover slip chamber, optical microscope images of the excess suspended particles were taken with 10× and 40× objectives in bright-field Köhler illumination. Images were taken during the dissolution process and after the 24-h equilibration to capture the changes during the critical stages of polymorphic conversion or new crystal growth. This also included in Tris-buffered salt solutions where morphologic changes were especially evident and were also followed in real time under diffusion-controlled conditions.

#### Testing for polymorphs: heating a dry powdered sample

In many of the dissolution experiments, the Yomesan powder dissolved, reached a maximum, but then showed a decrease in supernatant concentration, especially after overnight stirring. Thus, suspecting that the Yomesan tablet materials contained one or more polymorphs, 100 mg of dry powder was placed in a 20 mL dry scintillation vial equipped with a thermocouple positioned in the powder at the bottom of the vial. The glass vial was warmed on a heating plate at a few degrees per minute to ~ 100 °C, held for a few minutes, then warmed further to ~ 200 °C, and held at this temperature for 15 min. This experiment was an initial attempt to remove the waters of crystallization of the monohydrates and followed the reports by de Villers et al. (2004) and van Tonder et al. (2004) who measured dehydration transitions for H_A_, at 102 °C, and H_B_, at 174 °C. A cover-glass placed over the top of the vial detected the release of water at these two temperatures, indicating their transitions. This dehydrated material was then tested in a dissolution experiment.

## Results

Results are now presented along with some relevant discussion of the results. The “Discussion” section focuses mainly on the outcomes and impact of the discoveries made.

### Concentration dependence of 300 µM, 600 µM, and 1 mM Yomesan-niclosamide in pH 9.35 Tris buffer

#### Yomesan contains a UV-Vis-blocking “impurity”

The main issue discovered with Yomesan concerning the accurate measurement of supernatant niclosamide was that, while the Tris buffer was the most appropriate blank, the Yomesan tablet powder contained an excipient, water-soluble vanillin. This was first discovered at the lower pHs and for a 1 mM Yomesan-Niclosamide sample where less niclosamide was initially dissolved and the excipient was at a relatively high concentration.

**Fig. 2:**
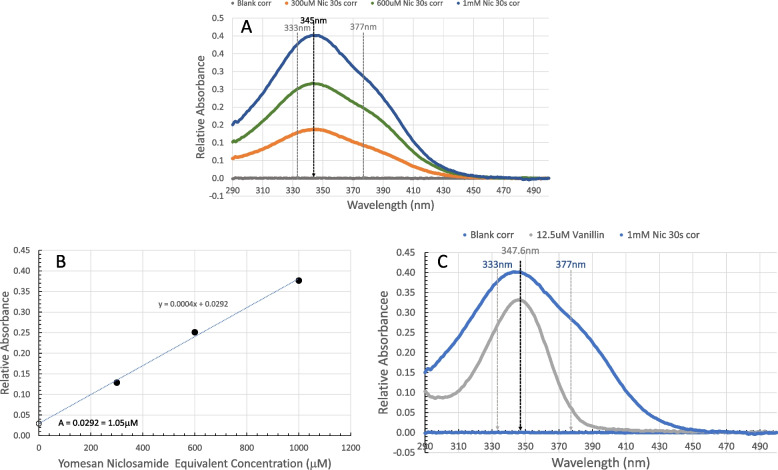
Comparison between baseline-corrected UV-Vis spectra for Yomesan at the 30 s time point for initial Yomesan niclosamide-equivalent concentrations of 300 µM, 600 µM, and 1 mM in pH 9.35 Tris buffer. **A** UV-Vis spectra showing the “impurity” peak indicated (black arrow) at 345 nm, and the expected double niclosamide peaks indicated (gray arrows) at 333 and 377 nm. **B** Relative absorbance at 333 nm vs. initial added Yomesan niclosamide-equivalent concentration (µM) reveals a linear dependence to the “impurity” absorbance with an intercept at an Absorbance of 0.0292 AU, which would be equivalent to a niclosamide concentration of 1.05 µM. **C** Comparison between the UV-Vis spectra for the 30s “impurity” for 1 mM Yomesan niclosamide and a 12.5 µM vanillin solution, both in pH 9.35 Tris buffer

**Fig. 3:**
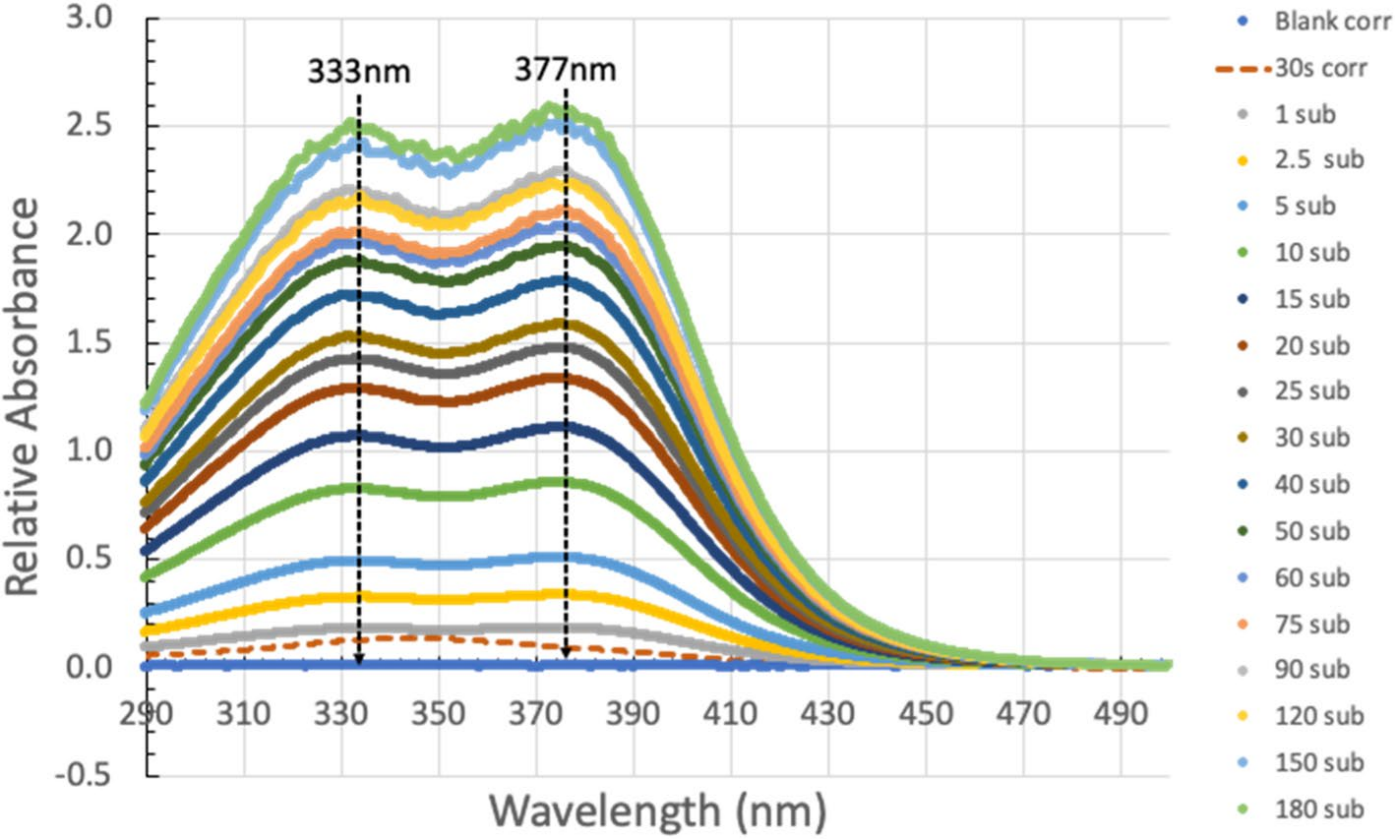
Baseline-corrected, 30 s subtracted spectra over 1–180 min dissolution for 300 µM fresh Yomesan niclosamide in nominal pH 9.35 Tris Buffer. The 30 s scan is shown as the dashed orange line and this 30 s scan was subtracted from each subsequent scan for 1 – 180 min. Each spectrum is an average of three scans (note: legend is ordered as 180 min to blank; graph is blank to 180 min)

To reveal the excipient absorbance from the niclosamide spectra (as shown later in Fig. 3, and in Additional file 1: Supplementary Information, S7. Comparative UV-Vis Spectra, Figs. S8 and S9), a UV-Vis spectrum was obtained at the first 30 s dissolution time point. This gave the soluble excipient “vanillin impurity” enough time to dissolve with minimal niclosamide absorbance. Thus, 300 µM, 600 µM, and 1 mM niclosamide-equivalent Yomesan powder (2.55 mg, 5.10 mg, and 8.33 mg respectively) were each added to 20 mL of Tris buffer at pH 9.35. Figure 2A shows the baseline-corrected spectra for each 30 s scan for initial Yomesan niclosamide-equivalent concentrations of 300 µM, 600 µM, and 1 mM in pH 9.35 Tris buffer.

As is clear from the spectra, the single λ_max_ absorbance peak at ~ 345 nm associated with the absorbing vanillin can cover up niclosamide’s two λ_max_ peaks at 333 and 377 nm and so interfere with the initial dissolution measurement of niclosamide. Dynamic light scattering (DLS) measurement (autocorrelation curve was equivalent to a filtered control) showed that this filtered solution did not contain any particles, and so the peak was not due to any suspended material.

This 30 s spectrum was subtracted from all subsequent time points for all Yomesan spectra. The slight “bump” at ~ 377 nm indicates that there was perhaps some slight niclosamide absorbance underneath this impurity spectrum. However, subtracting this 30 s peak is justified since, beyond this time, as shown in later dissolution spectra in Fig. 3 (and for all spectra in Additional file 1: Supplementary Information, S7. Comparative UV-Vis Spectra, Figs. S8 and S9), the characteristic double-peaked niclosamide spectrum starts to dominate, and so this is the best that can be done under the circumstances.

Plotted in Fig. 2B is the relative absorbance at 333 nm for the initial 30 s scans of the 300 µM, 600 µM, and 1 mM Yomesan niclosamide-equivalent concentrations. If uncorrected for, these absorbance values would represent significant errors in the niclosamide concentration of 9.6 µM, 17.8 µM, and 26.1 µM, respectively. Interestingly, the *y*-intercept of the three data points is 0.0292 A. This would represent a niclosamide concentration of just 1.05 µM and so supports the approach of subtracting this 30 s, largely impurity, spectrum.

Shown in Fig. 2C is a comparison between the UV-Vis spectra for the 30 s “impurity” for 1 mM Yomesan niclosamide and a 12.5 µM vanillin solution, both in pH 9.35 Tris buffer, and confirms that vanillin is the “impurity” (see Additional file 1: Supplementary Information, S2 for full vanillin spectra and concentration dependence in Figs. S2 and S3).

#### Niclosamide is readily extracted into pH 9.35 Tris buffer from Yomesan tablet powder at niclosamide-equivalent concentrations of 300 µM, 600 µM, and 1 mM

Data is now presented for the dissolution of Yomesan powder into a series of niclosamide-equivalent total concentrations of 300 µM, 600 µM, and 1 mM. As described above, the spectra were influenced by the presence of vanillin, that was subtracted, and the 333 nm peak was used to provide plots of the supernatant niclosamide concentration versus time after addition of Yomesan powder. Taking the absorbance values at 333 nm and converting them to niclosamide concentrations using a UV-Vis calibration provides a plot of supernatant niclosamide concentration (µM) versus time after addition of the Yomesan powder (mins) for each of the Yomesan-niclosamide equivalent concentrations of 300 µM, 600 µM, and 1 mM. While similar in form, taking these concentrations in series allows their individual features to be presented. First though briefly consider dissolution theory.

##### Noyes–Whitney

The approach to equilibrium saturation concentration for the dissolution of particulate material for a mass of drug (*m*) dissolved over time (*t*) is represented by the differential Noyes–Whitney equation (Hattori et al. 2013),

$$\mathrm{dm}/\mathrm{dt}\;=\;\mathrm{KS}({\mathrm C}_{\mathrm s}-{\mathrm C}_{\mathrm t})\mathrm{dt}$$

where *K* represents the constant conditions associated with the diffusive process. It contains *D*, the diffusion coefficient of the substance; *V*, the volume of solution; and *h*, the thickness of the diffusion layer for a given stirring rate; *S* is the surface area of the exposed powdered particles; *C*_*s*_ is the saturated concentration of the drug in the supernatant; and *C*_*t*_ is its concentration at time t.

The conditions (*D*, *V*, *h*) and stirring rate (300 rpm in a 20 mL vial) were essentially the same for all samples. While the small amount of sampling removed a small amount of suspended powder, this powder was removed in equal aliquoted volumes, and so the ratio of V and S was kept relatively constant, and the remaining excess powder was allowed to dissolve. The only other variable is the saturation concentration which, as reported before, is pH-dependent (Needham 2022). With a nominal pH of 9.35 and final pH of 9.26 at 180 min, the pH of the supernatant solution was relatively constant throughout the dissolution. Thus, integrating and taking logs shows that Log (C_t_ –C_s_)/C_s_ is proportional to t, and plotting the supernatant niclosamide concentration versus time should give a logarithmic approach to equilibrium.

##### 300 µM Yomesan-niclosamide equivalent

With the impurity accounted for, Fig. 3 gives the baseline-corrected, 30 s subtracted spectra over 1–180 min, for the lower 300 µM Yomesan-niclosamide concentration in nominal pH 9.35 Tris buffer. This kind of spectral series was obtained for all samples and typically shows the vanillin impurity (dashed line). (For all spectra, see Additional file 1: Supplementary Information, S7. Comparative UV-Vis Spectra, Figs. S8 and S9.)

**Fig. 4:**
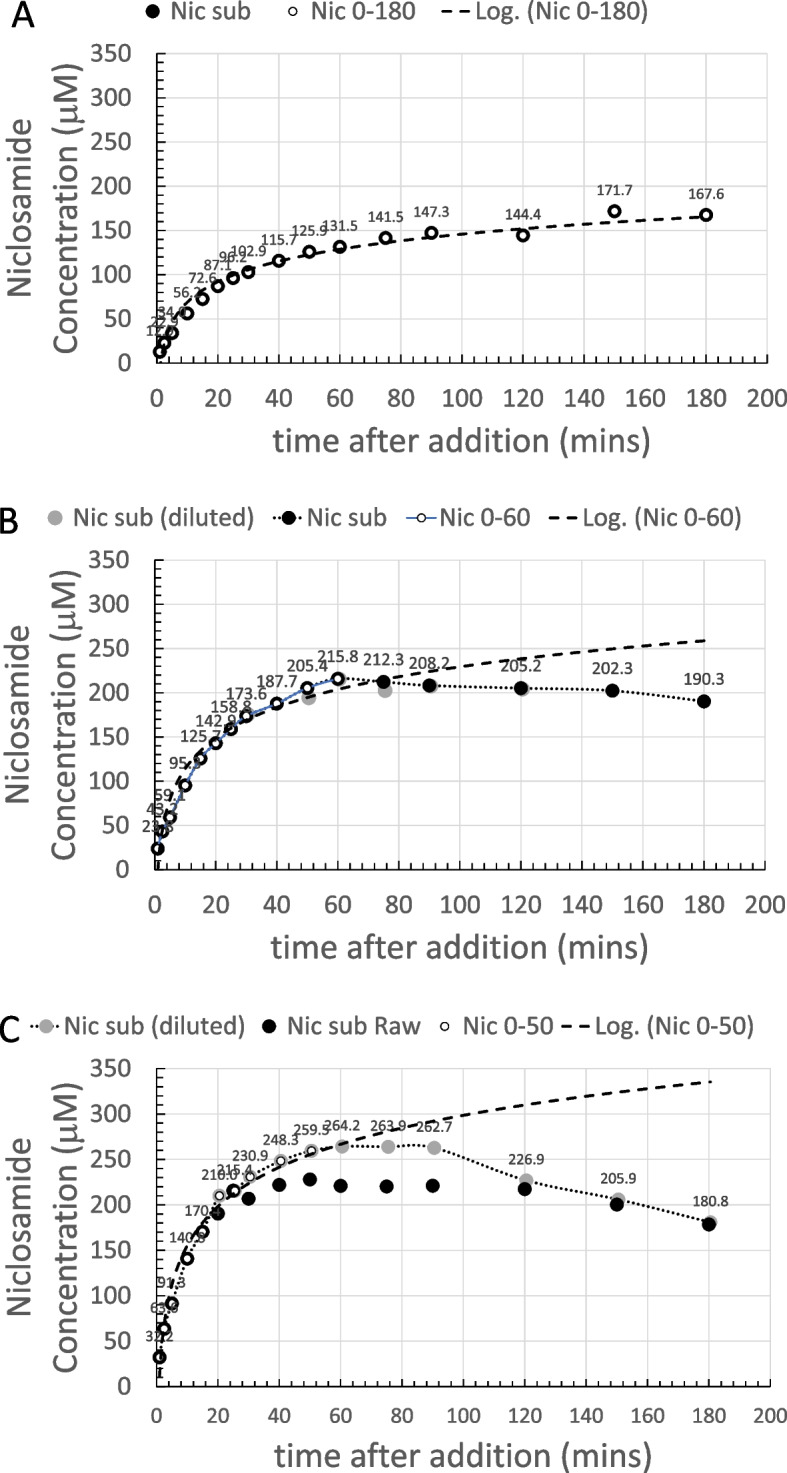
Dissolution of Yomesan-niclosamide equivalent in nominal pH 9.35 Tris buffers (0–180 min): **A** 300 µM, **B** 600 µM, and **C** 1 mM. White symbols signify the data points used for the logarithmic fit (dashed line) up to each peak time point of 180 min, 60 min, and 60 min respectively. Gray-filled symbols (**B** and **C**) represent a 2× dilution as the niclosamide concentration increased and approached the limit of detection for the UV-Vis absorbance detector. Error bars were ~ 1.5 µM and so within the filled symbols. The dotted line is to guide the eye and show the deviation from logarithmic

The final pH at 180 min was pH 9.27 and so had been reduced slightly compared to the initial nominal pH, possibly because of CO_2_ absorption into solution and niclosamide itself is a weak acid. The 30 s scan is shown as the dashed orange line. This 30 s scan was subtracted from each subsequent scan for 1–180 min and revealed the characteristic double peaked UV-Vis profile. Each spectrum is an average of three scans; their overall intensities increased with time as dissolution proceeded. The data for 333 nm is shown next along with the other concentration data in Fig. 4.

For the 300 µM sample (Fig. 4A), dissolution of niclosamide proceeds, as expected, in a logarithmic fashion. The white-filled symbols within the black-filled symbols signify the data points that were used for the logarithmic fit. In this case, all the data out to 180 min fitted a logarithmic dissolution. For the 300 µM sample, the supernatant niclosamide concentration reached a peak of ~ 170 µM at the 2.5−3 h time point.

While this data does fit a logarithmic dependence as the concentration approaches relative equilibration at 180 min, this would not necessarily be the case for the higher concentrations and especially at longer times (on day 2) when polymorphic changes occurred, and total solubilities concomitantly decreased.

##### 600 µM Yomesan-niclosamide equivalent

As shown in Fig. 4B, after addition of the powdered material, dissolution of niclosamide from the excess powder again proceeded in a logarithmic fashion, but this time, only up to the first 60 min. The black symbols represent the raw data that at these high absorbances (~ 3.0 AU) were starting to become noisy (see Supplemental Information Fig. S8 B).

Samples were therefore diluted by a factor of 2× and remeasured, as represented by the gray-filled symbols. As can be seen, the noisy samples do average out to what was measured in the more acceptable spectrometer range, and so the limits of the instrument were approached but not exceeded. The supernatant niclosamide concentration reached a peak of about 216 µM, plateaued, and then started to decrease over the next 2 h to 190 µM at the 180 min time point, where the pH was 9.22.

The white symbols again represent the data points that were used for the logarithmic fit, showing that, based on the dissolution profile of the first 60 min, this initial material would have gone on to achieve a total solubility of ~ 250 µM at the 3 h time point. However, as shown by the dotted line to guide the eye, beyond 60 min, dissolution deviated from logarithmic and there was no further increase in the niclosamide concentration, and in fact, it decreased. The optical microscope images presented later (Figs. 12 and 13) reveal that, during the dissolution process, there was a slow growth of new crystalline material on the original excess, undissolved Yomesan-niclosamide particles. This appears to be the mechanism that reduces the supernatant niclosamide as it equilibrates with this new hydrated polymorphic material that is known to have a lower solubility than the anhydrate (van Tonder et al. 2004).

##### 1-mM Yomesan-niclosamide equivalent

For the 1 mM niclosamide equivalent, the absorbance data (black symbols) shown in Fig. 4C did reach the limit of the instrument at absorbances of 3.4 AU starting at ~ 50 min and then went beyond the acceptable spectrometer range (see Supplemental Information Fig. S8C). Therefore, the 2× diluted samples (gray symbols and dotted line to guide the eye) gave a more reliable measurement of niclosamide concentration. The initial nominal pH was 9.35 and at 180 min had reduced slightly to pH 9.22.

The supernatant niclosamide concentration peaked at ~ 264.2 µM, and the initial dissolution was heading for a logarithmic value of 350 µM, which is on the order of the solubility limit for the anhydrous AKSci niclosamide at pH 9.22 (Needham 2022). Thus, for this 1 mM dissolution profile, there was a rapid increase in supernatant niclosamide concentration with dissolution of the Yomesan powder up to a peak at ~ 60 min, and then a reduction in niclosamide concentration at the 180 min point to 180.8 µM, again reflecting a possible polymorphic change.

##### Summarizing the dissolution data at pH 9.35

**Fig. 5:**
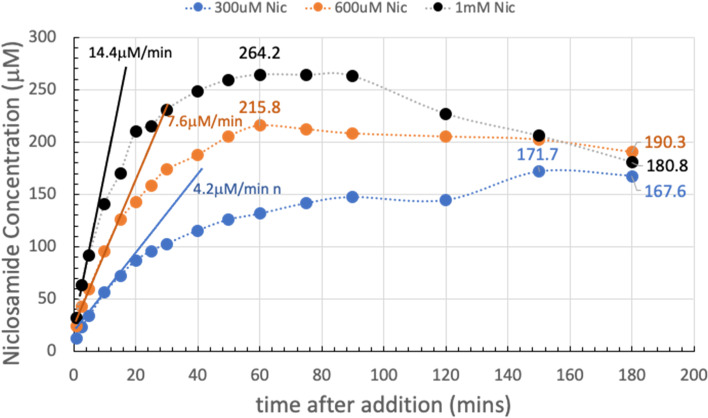
Summary of concentration data at pH 9.35 (0–180 min). Dissolution of Yomesan-niclosamide at niclosamide equivalents of 300 µM, 600 µM, and 1 mM in nominal 9.33 TB (final pHs ~ 9.2). 180-min time points average to 179.4 µM ± 11.4 µM. Initial rates of dissolution are also shown

It is now revealing and instructive to compare each of these dissolution plots on the same graph. The data from Fig. 4A, B, and C are summarized in Fig. 5 for each of the 300 µM, 600 µM, and 1 mM Yomesan-niclosamide equivalent concentrations in nominal pH 9.35 Tris buffer (TB) at 23 °C. The plots in Fig. 5 show how the higher the amount of Yomesan-niclosamide powder available, the higher the peak mount of niclosamide dissolved. However, the peak amounts (yields) of niclosamide dissolved do not necessarily increase proportionately with the amount of Yomesan powder initially added. This data is presented in full in Additional file 1: Supplementary Information, S4. Fraction of Niclosamide Extracted from Yomesan Powder and Fig. S5.

Briefly, maximum supernatant concentrations of niclosamide extracted by dissolution of Yomesan tablet powder in nominal pH 9.35 Tris buffer were achieved for the 300 µM, 600 µM, and 1 mM at 3 h, 1 h, and 1 h, respectively. These were 171.7 µM, 215.8 µM, and 264.2 µM and so represented extraction yields of 57.2%, 36.0%, and 27.4%. Interestingly, none of the dissolutions, and especially the 1 mM niclosamide equivalent (with ~ 3× excess material), did not quite reach the expected pH-dependent concentration of 318 µM that was achieved for a pure niclosamide sample of the anhydrous AK Sci niclosamide at the same pH of 9.22 (Needham 2022).

While the lower concentrations dissolved more slowly (smaller total surface area of the lower amounts of powdered material), it might be expected that they should all attain the same peak solubility. However, this was not the case and so there are confounding processes at work to limit the final concentration and even reduce it upon continued stirring. While excipients may bind and hold niclosamide, the main processes seemed to be the growth of a lower solubility monohydrate polymorph that depletes the soluble niclosamide in supernatant solution.

At the 180-min time point, supernatant niclosamide concentrations for each of the 300 µM, 600 µM, and 1 mM powdered Yomesan-niclosamide added were 190.3 µM, 180.3 µM, and 167.6 µM respectively, giving an average 180 min-value of 179.4 µM ± 11.4 µM, and attained approximately the same final pH of ~ pH 9.2.

It is also clear from the solid straight lines in Fig. 5 that the initial rates of dissolution increased as more powder was available. As quantified in Additional file 1: (Supplementary Information, S5. Initial Rates of Dissolution, Fig. S6), initial rates over the first 5, 10, and 15 min were 14.4 µM/min for 1 mM; 7.6 µM/min for 600 µM; and 4.2 µM/min for 300 µM, respectively. Thus, we can expect that the more niclosamide available the faster it will increase in supernatant concentration. However, as also shown above (and in Fig. S5), there is a diminishing return of the yield with increased amounts added. An optimized extraction protocol might therefore actually take the lower initial added amount of Yomesan powder to obtain the highest yield. How this relates to a scaled-up extraction protocol will be demonstrated at the end of the paper (see A Scaled-Up Example).

#### Day 2 data: an overnight stir results in a decrease in the supernatant niclosamide concentration

**Fig. 6:**
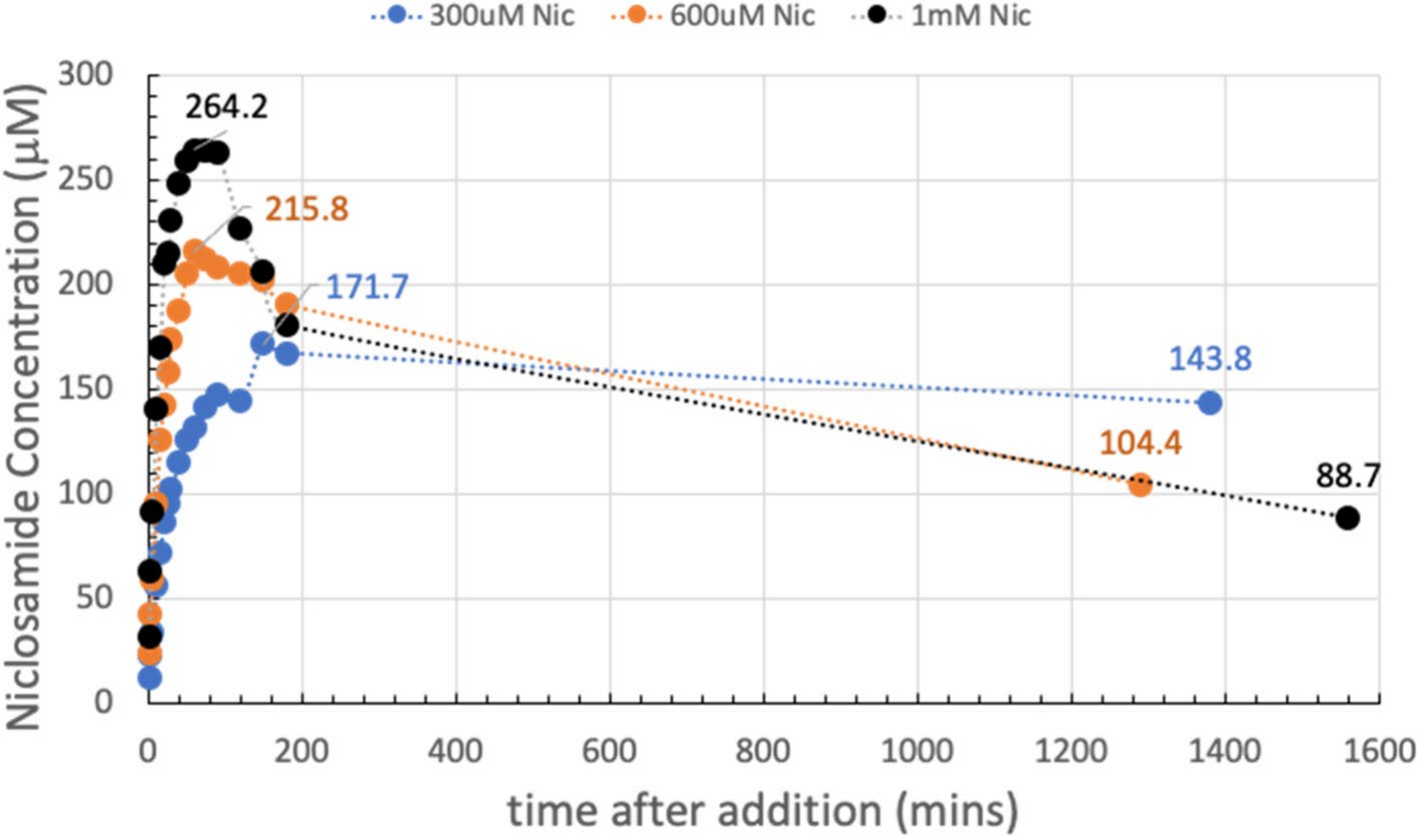
Yomesan-niclosamide equivalent concentrations of 300 µM, 600 µM, and 1 mM in nominal 9.34 TB (0–day 2). Final pHs were ~ 9.2. Peak concentrations: 171.7 µM, 215.8 µM, and 264.2 µM; day 2 concentrations: 143.8 µM, 104.4 µM, and 88.7 µM respectively; average day 2 value = 112.3 µM ± 28.4 µM

The samples in Fig. 4 were each stirred overnight in order to allow enough time for the supernatant, that was still in contact with the suspended excess powdered material, to approach an equilibrium niclosamide concentration. Figure 6 shows the dissolution of Yomesan tablet material at the Yomesan-niclosamide-equivalent concentrations of 300 µM, 600 µM, and 1 mM in nominal 9.35 TB measured from 1 min to day 2.

As is clear from each of the plots in Fig. 6, the maximum peak concentrations for supernatant niclosamide (171.7 µM, 215.8 µM, and 264.2 µM), were reduced on day 2 after the overnight stir to 143.8 µM, 104.4 µM, and 88.7 µM, giving an average day 2-value of 112.3 µM ± 28.4 µM.

This experiment therefore showed that niclosamide can be successfully extracted from crushed powdered Yomesan at pH 9.35 in Tris buffer. In order to provide the highest concentrations, the dissolution suspensions should be filtered to remove the equilibrating excess powder after ~ 1 h of dissolution for a 600 µM and 1 mM initial concentrations while dissolution can proceed for 3 h (as shown later in the scaled-up experiment Fig. 21). Otherwise, the supernatant concentrations decrease due to the conversion and crystal growth of the lower solubility hydrates.

### Making and testing the Yomesan anhydrate

#### Testing for polymorphs by heating a dry powdered sample

Suspecting that Yomesan already contained some of the lower solubility monohydrate, 100 mg of Yomesan powder was warmed in a 20 mL scintillation vial equipped with a thermocouple placed in the powder and heated slowly on a heating plate. Temperature and time were determined during the heating process and (in the absence of access to a thermogravimetric analysis (TG)) a glass coverslip was placed over the mouth of the vial to collect any condensed water of crystallization that was expected. As reported by van Tonder et al., using DSC and TG, only one endotherm (melting) is observed in the DSC trace of the anhydrate (onset 219 °C and a peak maximum at 229 °C). However, depending on the heating rate (5, 10, 20 °C/min) niclosamide monohydrate H_A_ has a dehydration transition at 102 °C, 112 °C, and 125 °C respectively, while HB dehydrates at 174 °C, 178 °C, and 184 °C.

**Fig. 7:**
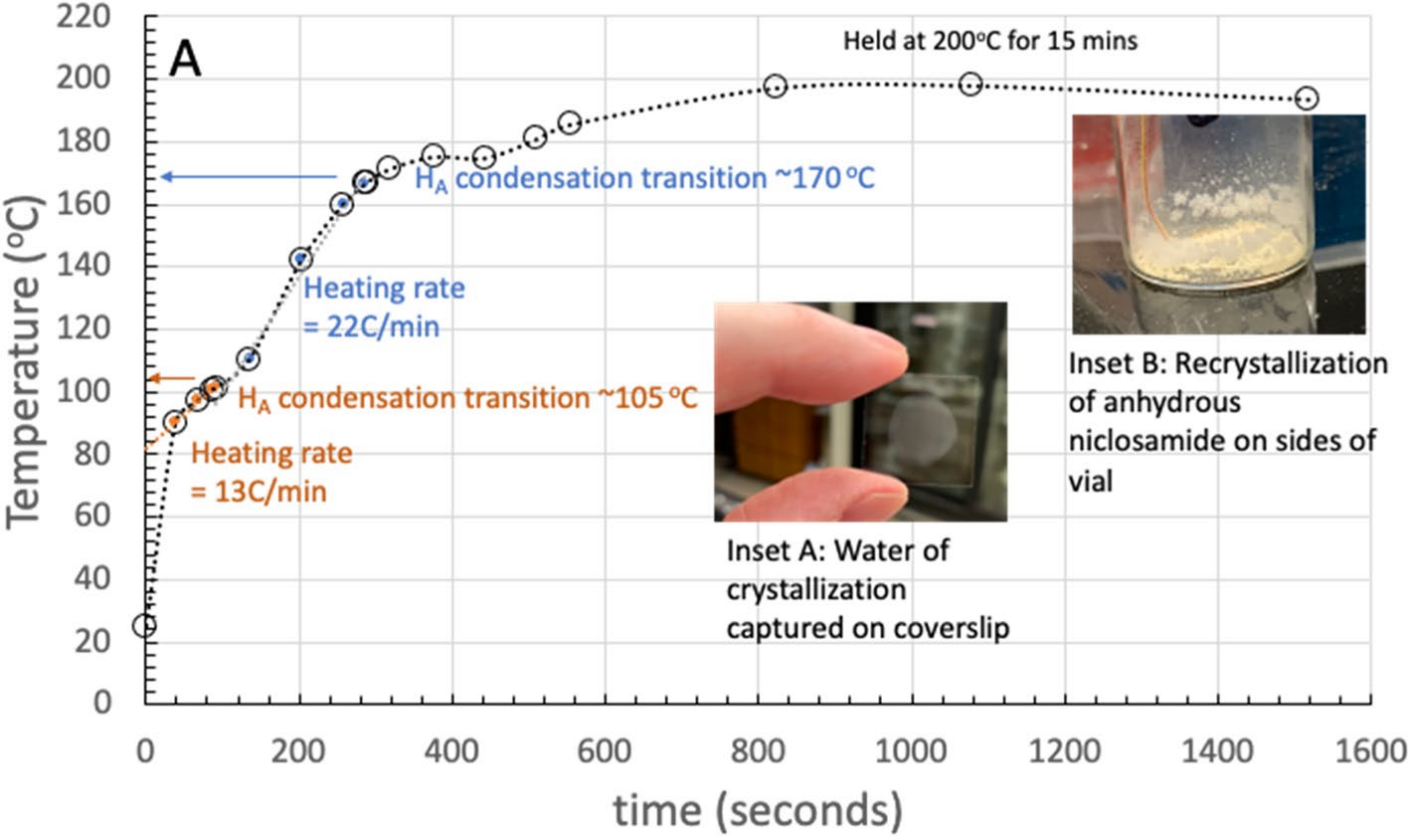
Experimental set up for heating 100 mg of Yomesan powder warmed in a 20 mL scintillation vial equipped with a thermocouple, placed in the powder, and heated slowly on a heating plate. Heating rates and H_A_ (orange) and H_B_ (blue) transitions each observed by condensation on a cover glass (Inset A). Held at 200 °C, niclosamide appeared to recrystallize on the sides of the vial (Inset B)

In this preliminary experiment carried out to observe the water loss by vapor-condensation, as shown in Fig. 7, the sample was initially heated at 13 °C/min and the first condensation transition was observed on the cover slip (Fig. 7 inset A) at ~ 105 °C, after which the heating was continued at 22 °C/min and a second condensation was observed as the temperature approached 170 °C.

Heating was continued, and the sample was held at ~ 190–200 °C for another 15 min in an attempt to dehydrate the Yomesan powder and obtain the anhydrous niclosamide material, as had been done and shown by van Tonder and de Villers (de Villiers et al. 2004; van Tonder et al. 2004).

On being held at 200 °C, as also shown in Fig. 7 inset B, there was some recrystallization on the sides of the vial as well as in the powder itself. This indicates that niclosamide could possibly sublime and recondense-recrystallize as presumably the anhydrate. See later Fig. 15 in the section on microscopy for microscope images of these dry crystals.

#### Dissolution of the Yomesan anhydrate

**Fig. 8:**
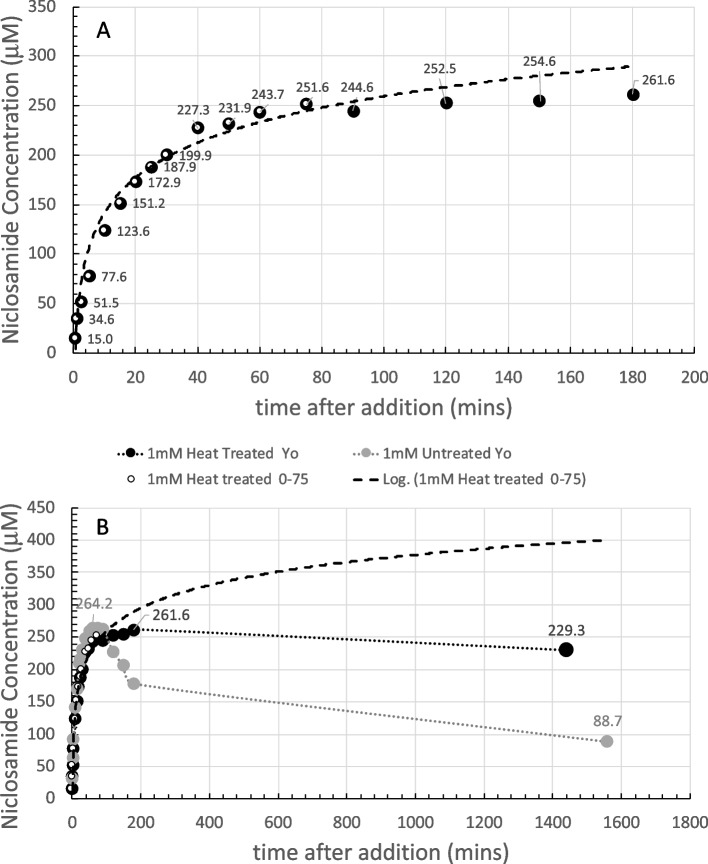
Dissolution of ~1 mM 200 °C heat-treated Yomesan in nominal pH 9.35 Tris buffer at 23 °C: **A** 1 to 180 min, final pH 9.32 with logarithmic fit to initial data (dashed line); **B** comparison between heat-treated Yomesan (black-filled symbols) and untreated Yomesan (gray-filled symbols) from Fig. 4C in 9.35 TB vs time after addition out to day 2 final pH 9.26. Dotted lines are to guide the eye

Having dehydrated the Yomesan powder, the dissolution experiment was repeated with this 200 °C heat-treated sample, as shown in Fig. 8.

The same protocol was used as above; ~1 mM Yomesan niclosamide-equivalent was added to 20 ml of stirred nominal pH 9.35 Tris buffer and supernatant samples were taken to provide the dissolution profile at 23 °C.

Figure 8A shows the 1–180 min of dissolution on day 1 and, in contrast to the profile in Figs. 4C, 5, and 6, for the untreated Yomesan, the supernatant niclosamide concentration did not peak and decrease. Rather, it simply continued to increase following a logarithmic dissolution reaching 261.6 µM at the 3 h time point with a slight reduction in pH to pH 9.32. Following the overnight stir at 24 h, Fig. 8B shows that for this heat-treated sample there was eventually a slight reduction in supernatant niclosamide concentration to 229.3 µM at a final pH of 9.26. While the peak concentrations are very similar for the heat treated and untreated Yomesan (261.6 µM and 264.2 µM respectively), the supernatant niclosamide concentration for the fresh but untreated Yomesan sample, also shown in Fig. 8B, reduced to 88.7 µM after the overnight stir.

Again, the reasons for these differences appear to be the state of the original powder, whether it contained mostly anhydrous material (heat treated) or a mixture with H_A_ and H_B_ polymorphs (untreated) and the degree to which a certain sample converts to, or grows, additional hydrated crystals. Thus, having removed (most of) the H_A_ and H_B_ polymorphs that were present in the as-bought tablets by the heating protocol to 200 °C, and including what appeared to be even recrystallized anhydrate in the powder, the tendency to form and grow the hydrated crystals was very much lessened. The logarithmic dissolution for the first 3 h was actually heading towards ~ 400 µM on day 2 which depending on the pH again would be similar to the previously measured solubility of an anhydrous form of niclosamide at a similar dissolution pH (pH 9.32 gave 400 µM) (Needham 2022). Thus, heat treatment of the Yomesan niclosamide appears to return the anhydrate as reported by van Tonder et al. (2004) and de Villers et al. (2004), and this is reflected in the relatively high total supernatant niclosamide concentration that is obtained after dissolution of excess powder in nominally pH 9.35 Tris buffer.

### Extraction of niclosamide from Yomesan into pH 7.41, 8.35, 8.85, and 9.35 Tris buffer at 600 µM equivalent niclosamide

This second specific aim investigated the pH dependence of the dissolution and extraction process in order to further optimize the extraction of niclosamide from Yomesan tablet material. While it was not necessarily expected that the extracted niclosamide would reach the solubility limits reported earlier (Needham 2022), the hypothesis was that in an excess of the anhydrous component, niclosamide would dissolve, as shown above at pH 9.35, and that lowering the pH would reduce the amount in solution in a pH-dependent fashion. It was also hypothesized that the Yomesan niclosamide would again show a conversion to the lower solubility hydrate upon long-time exposure to the aqueous hydrating buffers. The goal, therefore, was to optimize the extraction at a reasonable initial concentration and check the pH dependence.

#### Yomesan-Niclosamide dissolution: pH 7.41 to 9.35

**Fig. 9:**
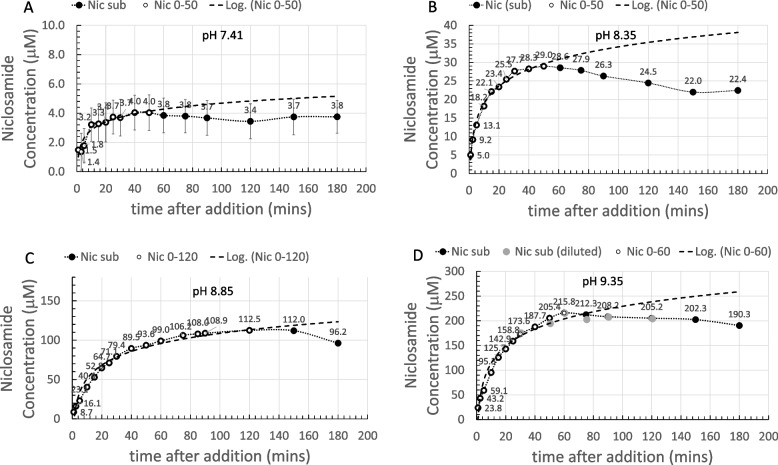
Dissolution of Yomesan-niclosamide equivalent in Tris pH buffers (0–180 min, black-filled symbols): **A** 300 µM, niclosamide nominal pH 7.41, final pH 7.31; **B** nominal pH 8.35 final pH 8.25; **C** nominal pH 8.85, final pH 8.77; **D** nominal pH 9.35, final pH 9.27 (B, C, D are at 600 µM niclosamide); White symbols signify the data points used for the logarithmic fit (dashed lines) up to each peak time point of 50 min, 50 min, 120 min, and 60 min, respectively. Gray-filled symbols (pH 9.35) represent a 2× dilution as the niclosamide concentration increased and approached the limit of detection for the UV-Vis absorbance detector. Error bars were ~ 1.5 µM and so within the filled symbols. The dotted line is to guide the eye and show the deviation from logarithmic. The day 2 concentration were 3 µM, 12.9 µM, 35.1 µM, and 112.3 µM (data not shown)

A series of 20 mM Tris buffers were made up to give nominal pHs of 7.41, 8.35, 8.85, and 9.35. The total niclosamide concentration was chosen to be 300 µM for pH 7.41, and 600 µM for pHs 8.35, 8.85, and 9.35. As seen earlier (Fig. 4B) 600 µM Yomesan-niclosamide in pH 9.35 would provide enough niclosamide to dissolve to its solubility limit, but the amount in solution would not completely exceed the upper limits of the UV5Nano. Data for each of the pHs is shown in Fig. 9. Because of the wide range of values (on the *y* axis), each graph is shown separately and discussed individually.

At the lowest nominal pH 7.41 (Fig. 9A), a 300 µM sample was selected in order to reduce the amount of impurity present while still providing an excess of Yomesan-niclosamide for dissolution. The spectra were nevertheless dominated by the vanillin signal (as shown in Supplemental Information S7 Comparative Spectra, S7.2 pH Dependence, Fig. S9 A). However, as before, subtracting the 30 s spectrum successfully revealed the characteristic double-peaked profile of niclosamide. The first 50 min adhered to a logarithmic dissolution, and as expected, achieved a peak supernatant niclosamide of only 4 µM. The niclosamide concentration essentially plateaued over the next 2 h.

The final level of dissolution at 180 min was similar to the earlier measurements of pure niclosamide (Needham 2022) that were in the 3–4 µM range at this final pH 7.31. This data underlines the usual fears in the literature about niclosamide having such a low solubility at neutral pH or in deionized water. The day 2 concentration was 3 µM at pH 7.31.

At pH 8.35 Fig. 9B), the supernatant niclosamide concentration rose to 29.0 µM at 50 min, showing a logarithmic dissolution up to this point, followed by the usual reduction in concentration to 22.4 µM at the 3 h time point where the pH was 8.25. The day 2 concentration was 12.9 µM (see also Additional file 1: Supplementary Information S7 Comparative Spectra, S7.2 pH Dependence, Fig. S9B).

At pH 8.85 (Fig. 9C), the peak concentration was 112.5 µM and the logarithmic dissolution went all the way out to 120 min. This was followed by a slight reduction in concentration to 96.2 µM at the 3 h time point where the pH was 8.77. The day 2 concentration was 35.1 µM (see also Additional file 1: Supplementary Information S7 Comparative Spectra, S7.2 pH Dependence, Fig. S9C).

Finally, at pH 9.35, as shown earlier (Fig. 4B), and reproduced here, the peak concentration reached 215.8 µM at 60 min, reducing to 190.3 µM at pH 9.27 at the 3 h time point. These peak concentrations represent the dissolution of mainly fresh anhydrous niclosamide, i.e., before any reductions due to the crystal conversions and growth. The day 2 concentration was 112.3 µM. Again, the resolution of the spectrophotometer was approached but not exceeded (see also Additional file 1: Supplementary Information S7 Comparative Spectra, S7.2 pH Dependence, Fig. S9D).

#### Summary plot with pHp theory

**Fig. 10:**
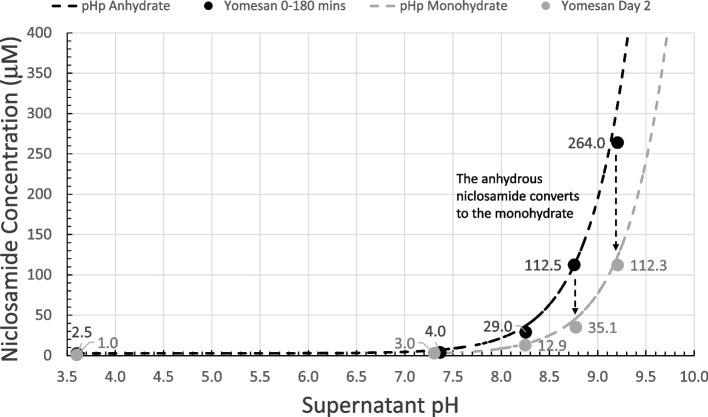
Yomesan-niclosamide dissolution as a function of Tris buffer pH: Maximum niclosamide concentrations from Fig. 9 (filled black circles) vs. supernatant pH, plotted along with pHp theory determined earlier (Needham 2022) (dotted black line), for anhydrous (AK Sci) niclosamide (pKa 7.12, S_w_ = 2.53 µM). Also shown are the day 2 equilibrated concentrations (gray-filled circles) and pHp theory for the monohydrate (dotted gray line) (*S*_w_ = 1 µM) (*S*_w_ = intrinsic solubility of the protonated niclosamide acid)

The peak concentrations (--data from Fig. 9) are plotted in Fig. 10 as a function of Tris buffer pH from nominal pHs of 7.41 to 9.35. The maximum supernatant niclosamide concentrations (black-filled symbols) show the expected increasing concentration with increasing pH. Also, plotted as a black-dashed line is pHp theory determined earlier (Needham 2022), for a niclosamide with a pKa of 7.12, and an intrinsic solubility for its the protonated niclosamide acid S_w_ of 2.53 µM. Since these maxima represent the dissolution of largely the anhydrous component of the added powder, it is satisfying that for crushed tablet material even in the presence if excipients data are very consistent with theory and previous data on a pure anhydrous (AK Sci) niclosamide material.

Allowing the suspensions to equilibrate by stirring over night for ~ 24 h shows the characteristic reductions in supernatant concentrations, plotted as gray-filled symbols. As can be seen, these equilibrated concentrations match the pHp theory for an equilibrated monohydrate (Needham 2022) for the same pKa of 7.12 and where the intrinsic solubility of S_w_= 1 µM (gray dashed line) that was again measured previously for pure niclosamide (Needham 2022).

What this summary plot nicely shows is that the initially, mostly anhydrous, Yomesan-niclosamide converts to the mostly monohydrate with overnight stirring. And so, in order to obtain the highest concentration of supernatant niclosamide, samples of Yomesan tablets should be allowed to dissolve for 60 to 180 min and then filtered to remove the equilibrating excess material.

### Morphologic assessment of the dissolution of Yomesan-niclosamide by optical microscopy

Evidence for the anhydrous to hydrated conversion is now provided in the images obtained by optical microscopy. In order to bring some additional characterization to the dissolution process and attempt to explain the differences in levels of extraction of niclosamide from the tablet materials, optical microscope images were taken of each of the samples as dry powder and during and after the 2-day equilibration process. These data are also intended to motivate future X-ray diffraction and Raman, differential scanning calorimetry, and thermogravimetric analyses that would confirm and further examine the nature of both tablet niclosamide as well as pure niclosamide samples as initial dry powder, and for any of the converted polycrystalline crystalline forms.

#### Typical components in Yomesan

**Fig. 11:**
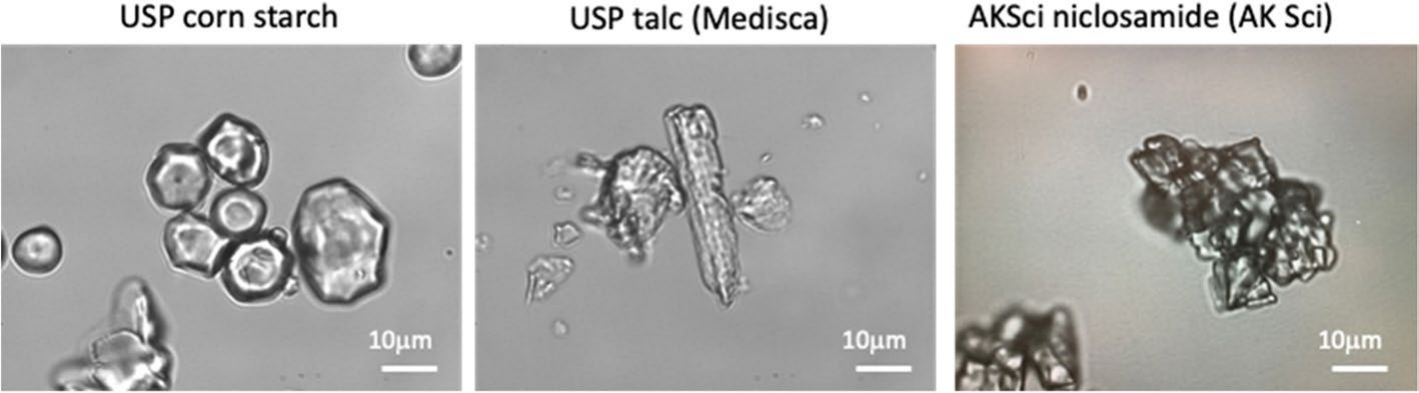
Optical microscope images of the main components of Yomesan powder: corn starch (Lab Alley, TX); USP talc (magnesium silicate) (Medisca); anhydrous pure niclosamide (AK Sci) as aggregates of small particles.  40× objective, scale bars are 10 µm, in bright field Kohler illumination optics (Nikon Diaphot)

Images of the typical components in powdered Yomesan were obtained to demonstrate that each are readily identified in the suspended materials. Figure 11 shows representative images of the three main tablet components that are expected to be present in the as purchased materials: corn starch, are mostly spheroid particles; talc, appears as larger elongated as well as particulate crystals depending on the processing; and pure niclosamide forms particulate aggregates, as from AK Sci.

#### Initial starting niclosamide particulate aggregates

**Fig. 12:**
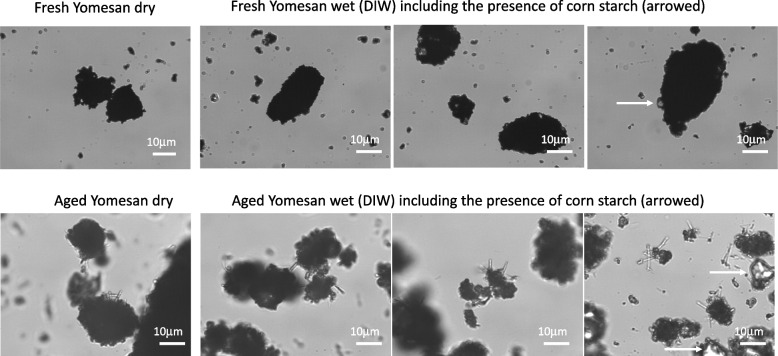
Optical microscope images of Yomesan powder: (top) a sample of featureless particulate aggregates of the fresh powder, and (bottom) crushed Yomesan aged several weeks at room temperature in a closed but frequently opened vial. Initial images (left) show dry material and (right) three images of the Yomesan powders suspended in deionized water. Needle-shaped crystals are evident on the aged particulate aggregates. Corn starch particles are indicated by arrows in the far-right image. 40× objective. Scale bars are 10 μm, in bright field Kohler illumination optics (Nikon Diaphot)

Shown in Fig. 12 are typical examples of the original ground-up Yomesan powders, fresh from the blister pack and an earlier sample that was exposed to ambient air and humidity for several weeks.

This earlier sample basically represents aged material where the tablet was obtained from the blister pack, crushed by mortar and pestle, and then stored for several weeks at room temperature in a closed glass vial, that was nevertheless opened to take samples for experimentation. What is evident from these images is that the fresh sample consists of niclosamide particle aggregates that can be ~ 10 to 50 µM in diameter and appear to be made up of smaller individual particles of ~ 1−2 µM in diameter. In this fresh sample (that was then stored under vacuum), there is a distinct lack of any other needle-like morphologies. In contrast, the aged sample had started to grow what appeared to be hydrated crystals of a characteristic needle-shaped H_A_ and H_B_ morphology. Corn starch particles are evident in the samples as indicated by the arrows in the far-right image.

Thus, the aged Yomesan powder appears to be a mixture of particulate aggregates and the needle morphology. This is reminiscent of what was seen previously (Needham 2022) with commercial supplies of pure niclosamide itself, where the higher solubility “AK Sci polymorph” was also comprised of featureless particulate aggregates, while the lower solubility “Sigma polymorph” was a mixture of aggregates and needle-shaped particles. This now provides new evidence and starts to explain the dissolution results, where the particle-aggregated morphology (anhydrate) showed a higher solubility across the whole pH range than that associated with the developing, more stable, needle-shaped, monohydrate polymorph.

#### Dissolution of fresh Yomesan-niclosamide does eventually convert to the hydrate

**Fig. 13:**
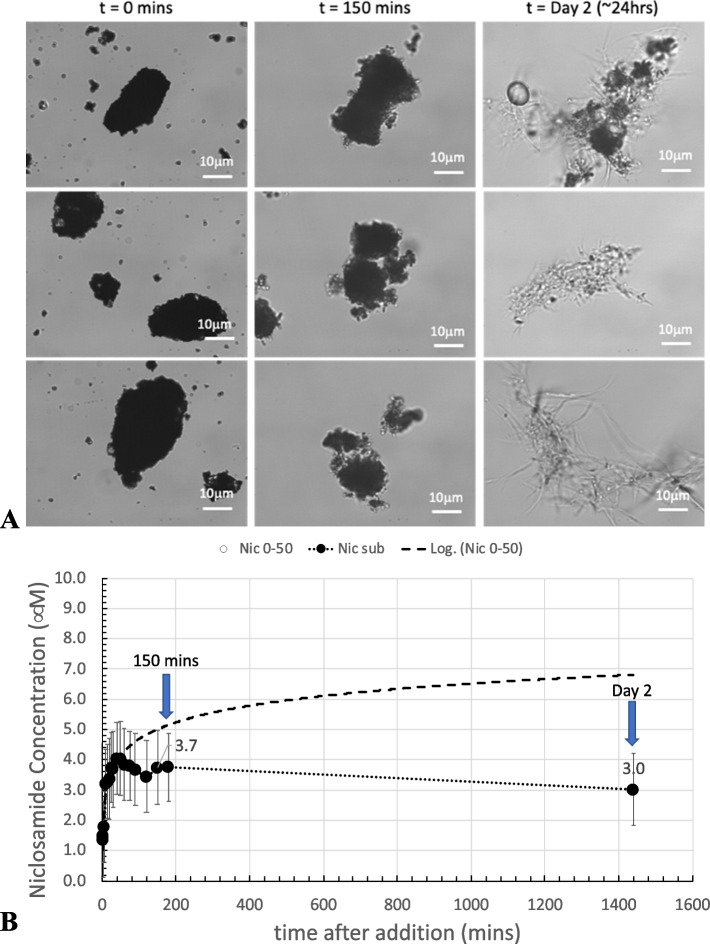
Dissolution of Yomesan at nominal pH 7.41. **A** Three typical optical microscope images of Yomesan-niclosamide particles at each time point, before (*t *= 0 min), during (*t* = 150 min), and after equilibration (day 2). 40× objective. Scale bars are 10 μm in bright field Kohler illumination optics (Nikon Diaphot). **B** Dissolution profile from Fig. 9A, extended to day 2

**Fig. 14:**
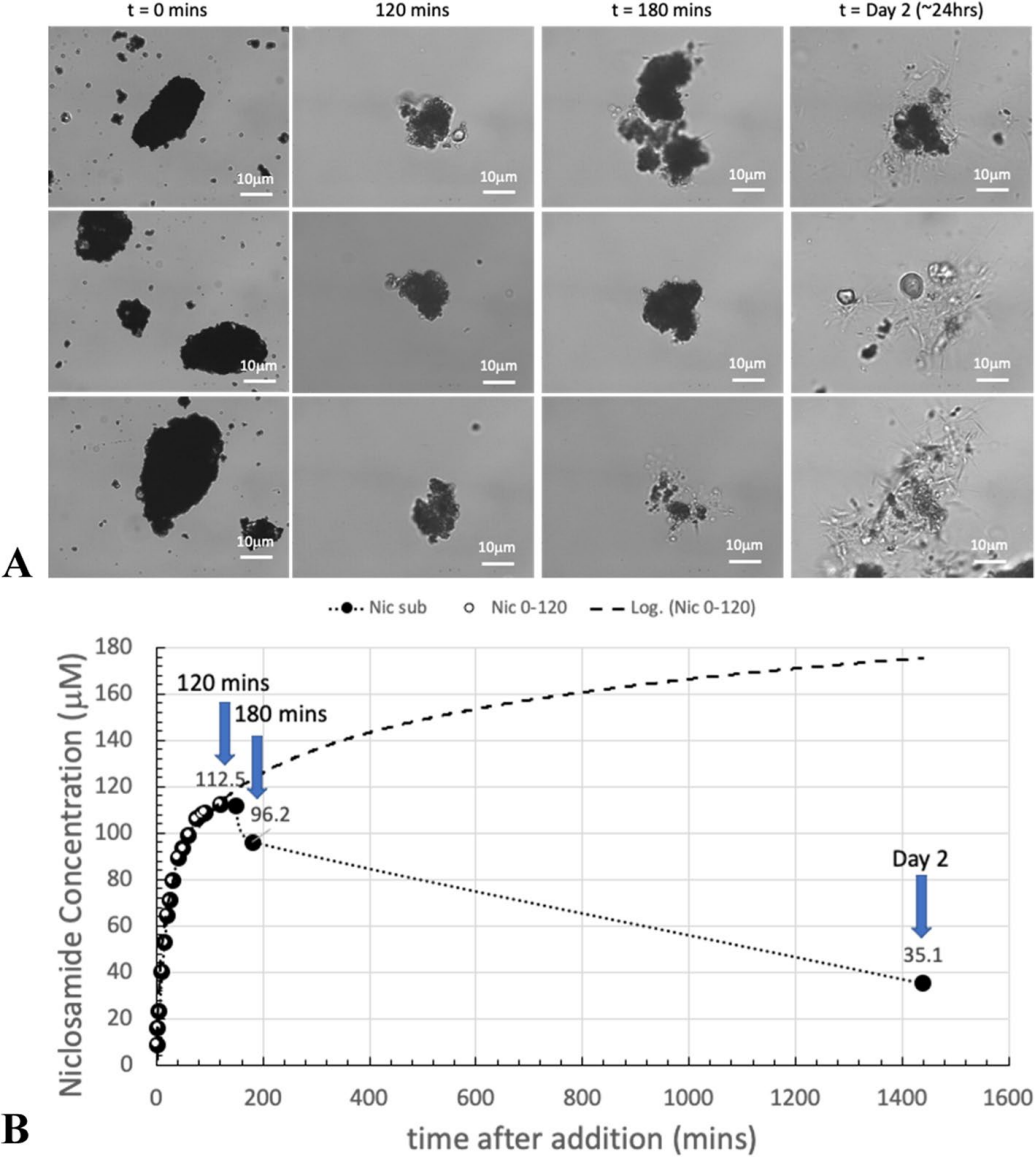
Dissolution of 600 µM Yomesan-niclosamide at nominal pH 8.85. **A** Optical microscope images of Yomesan-niclosamide particles before (*t* = 0mins), during (*t *= 120 min and 180 min), and after equilibration (day 2). **B** Dissolution profile from Fig. 8C, extended to day 2. 40× objective,. Scale bars are 10 μm in bright field Kohler illumination optics (Nikon Diaphot)

Presented next in Figs. 13 and 14 are a series of images of samples of the suspension taken before, during, and after the dissolution of Yomesan powders. The examples are from the pH dependence study above in Fig. 9 and extended to day 2. Regardless of concentration or pH, the initial, fresh, mostly anhydrous aggregates are seen to undergo a transformation as the particles in the aggregates start to grow more transparent outcrops and eventually needles. All of this coincides with the peak and then reduction in niclosamide supernatant concentration as seen in all of the above Yomesan dissolution graphs.

##### 600 µM Yomesan-niclosamide in nominal pH 7.41

Taking the nominal pH 7.41 sample from Fig. 9A, morphologically, in Fig. 13A, dissolution starts, as usual with the fresh, relatively featureless, particulate-aggregates.

As shown in Fig. 13B, dissolution proceeds and peaks at ~ 4.0 µM and then shows a slight decrease in supernatant niclosamide concentration at 150 min to 3.7 µM. At this point, the suspended particles have a slight change in morphology, with more transparent “outcrops” on the original aggregates that appear to be the beginnings of new crystal growth. This new growth coincides with the slight decrease in supernatant concentration to 3.7 µM.

The most dramatic change in morphology is seen on day 2, when the supernatant concentration had reduced to 3 µM and the whole sample shows a massive growth of needle-shaped crystals. These needles can be seen emanating from the original aggregates (Fig. 13A, top right) as well as forming stand-alone needle-masses (Fig. 13A middle and bottom right).

Thus, even though the concentration of niclosamide in suspension did not change by more than 1 µM, the particle morphology showed a dramatic conversion, by growth of what appears to be the hydrate needles.

##### 600 µM Yomesan-niclosamide in nominal pH 8.85

This conversion behavior was also seen at higher pHs and higher concentrations. Figure 14 shows the 600 μM Yomesan niclosamide in nominal pH 8.85.

In Fig. 14A, the relatively featureless initial particulate aggregates start to dissolve, and the supernatant concentration increases in Fig. 14B with dissolution to a peak of 112.5 µM at 120 min. At this point, in the suspension, the particles are not that changed. However, as dissolution and exposure to the buffered solution proceeds, the particles started to show some morphological conversion at 180 min when the supernatant niclosamide concentration had reduced to 96.2 µM. Then, with overnight stirring, the suspension acquired the now familiar needle morphologies emanating from the aggregate (Fig. 14A, top right), mixed needles and corn starch (Fig. 14A, middle right), or formed a mass of needles (Fig. 14A, bottom right).

By comparison, the other two tablet niclosamide samples (Lux and Niclosig) did not make such a conversion, and so it might be presumed that these tablets contained mostly, if not completely, anhydrous niclosamide.

Thus, when taken together, these images and the dissolution plots provide fairly convincing evidence that the niclosamide used in Yomesan is mostly anhydrous when fresh, can slowly convert to hydrated polymorphs on storage, and is especially sensitive to exposure to aqueous buffer. As shown above, within ~ 1 h of stirring excess powdered Yomesan tablet material in Tris buffers there is conversion and new crystal growth from already present hydrates mixed in with the original niclosamide used to make the tablets. This results in a decrease in the supernatant niclosamide concentration as it equilibrates with the new crystal growth of the lower solubility hydrate polymorph. These data on dissolution and images of morphological change again drive the further investigation of both tablet niclosamide as well as pure niclosamide samples by DSC, TG, Raman, and XRD.

#### Heat-treated Yomesan-niclosamide appears to recrystallize as the anhydrate

**Fig. 15:**
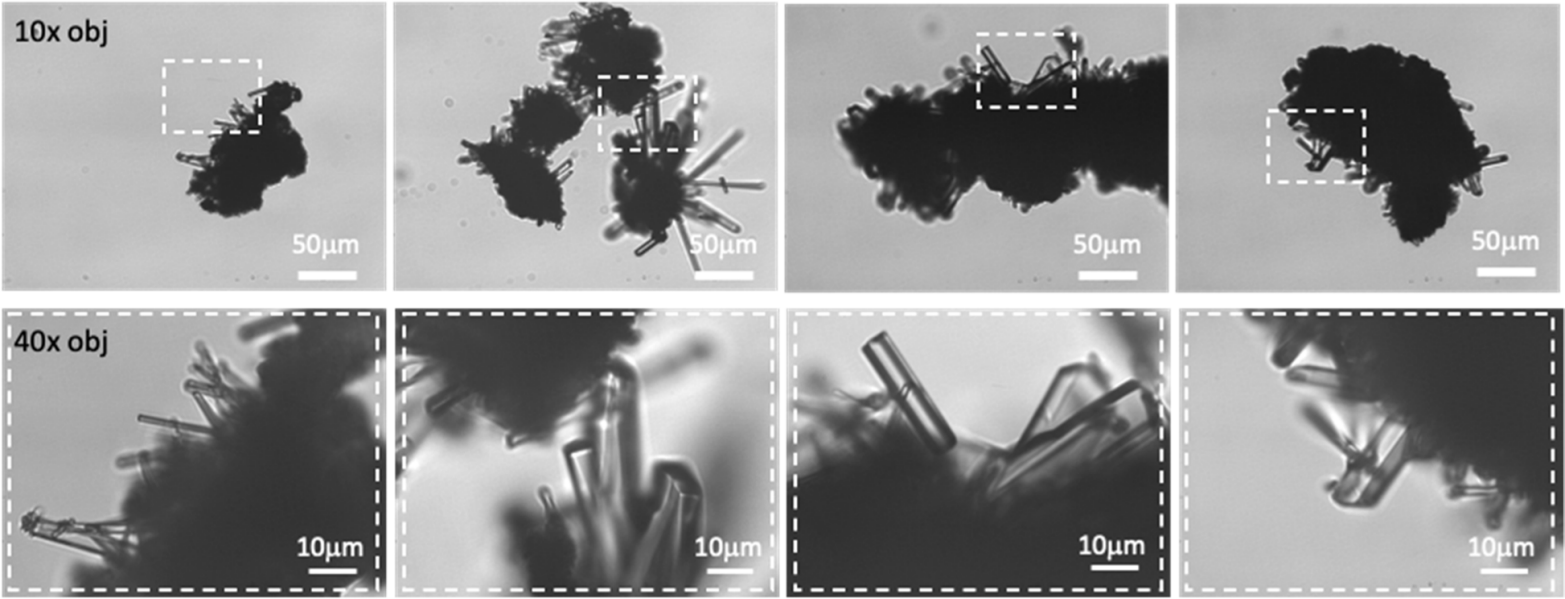
Optical microscope images of the dry heat-treated powder, showing, presumably, anhydrous recrystallization using 10× (top) and 40× (bottom) objectives. Regions of interest are shown at the two magnifications by the dashed boxes. Scale bars are 50 μm and 10 μm in bright field Kohler illumination optics (Nikon Diaphot)

When the 200 °C heat-treated anhydrous powder (as made in Fig. 7, and dissolved in Fig. 8) was observed in the optical microscope, there was extensive growth within the powder on the original particulate aggregates, of presumably the anhydrous niclosamide (Fig. 15).

As shown in Fig. 15, this recrystallization formed much larger and thicker crystal growth than seen for the monohydrates formed by growth in buffered solution. For example, comparing these images in Fig. 15 with the 40× images of the aged dry Yomesan-niclosamide in Fig. 11 and the growth during dissolution in Figs. 13 and 14. These observations again motivate more analytical DSC, TG, and XRD studies to confirm these new findings.

### Yomesan-niclosamide in Tris-buffered saline solutions (TBSS)

As mentioned earlier, the use of TBSS would provide a niclosamide solution of higher osmolality, so that it approached or matched the nasal osmolality of ~ 290 mOsm. However, as shown below in the picture of the niclosamide sodium vial in Fig. 17, equilibrating Yomesan-niclosamide in TBSS produced a rapid conversion of the undissolved excess powder material to a red-orange colored needle-like morphology. A standard 100 mM sodium chloride solution was chosen as the stock solution and Tris buffer (TB) was made at 20 mM according to the recipes at AAT Bioquest (AATbio.com 2021). Osmolalities for the Tris buffers were measured on an Osmometer (Advanced® Osmometer Model 3D3, Advanced Instruments, MA), therefore increased from ~ 40 mOsm for 20 mM TB, to 200 mOsm for the TBSS. Tests were done on Yomesan-niclosamide, the other tablet materials, and pure niclosamide. Only the Yomesan data will be shown here; the rest is reserved for a future publication that will include DSC, TGA, Raman, and XRD analyses to confirm this niclosamide sodium or co crystal composition.

#### Dissolution of 1 mM Yomesan-niclosamide in TBSS

Dissolution in Tris-buffered saline solution was carried out because of the potential need for an isotonic osmotically balanced solution of niclosamide, especially for a nasal spray. Salt solutions are routinely used as nasal hydrating solutions and are typically at ~ 290 mOsm. It was therefore important to check the dissolution of 1 mM Yomesan in nominally pH 9.23 Tris-buffered saline solution (TBSS) of 20 mM Tris buffer and 100 mM sodium chloride solutions, giving a hypo-osmotic solution of ~ 210 mOsm. (The thinking here is that a hypo-osmotic solution would help to osmotically drive the niclosamide solution into the mucosa up its water concentration gradient.)

**Fig. 16:**
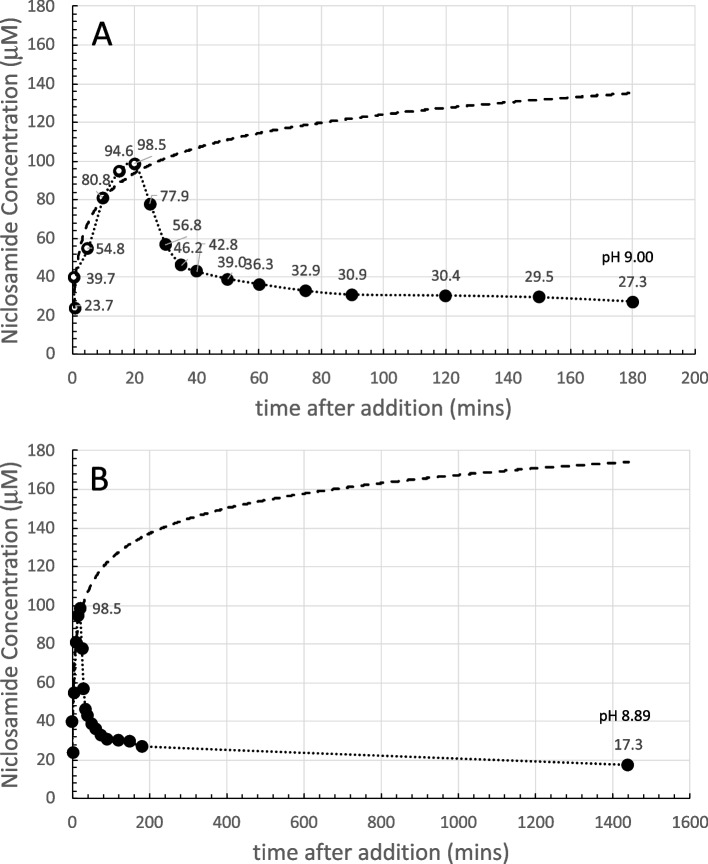
Dissolution of 1 mM Yomesan in nominally 9.23 Tris-buffered saline solution (TBSS): supernatant niclosamide concentration (µM) vs. time after addition (mins), baseline-corrected, 30 s corrected for, **A** 0–180 min. **B** Same data including day 2. Dashed line is the logarithmic fit to the initial (white symbol) dissolution; dotted line is to guide the eye

Surprisingly, as reported here for the first time, excess undissolved niclosamide was converted to a red, needle-shaped morphology in a matter of 30-min exposure to TBSS. This was accompanied by a quite different dissolution-concentration profile to that in just Tris buffer (TB). For example, the data presented earlier in Fig. 4C shows how a 1 mM Yomesan-niclosamide-equivalent concentration reached 264.2 µM in 1 h of dissolution in Tris buffer at a nominal pH 9.35. In contrast, in Fig. 16, a 1 mM Yomesan powder sample in a similar nominal pH of 9.23 but in Tris-buffered saline solution, only reached a peak of 98.5 µM at 30 min; it then rapidly decreased in supernatant niclosamide concentration.

At the 3 h time point, the niclosamide concentration was only 29.3 µM and dropped to 17.3 µM after 24 h of continual stirring, when the pH was still a relatively high, pH 9.0. This experiment was actually repeated six times with old and new Yomesan, all at nominal pHs of 9.23 to 9.35 achieving an average pH at equilibration of pH 9.1 ± 0.1 and an average day 2 supernatant concentration of 30.7 µM ± 10.6 µM. Data was also obtained for Yomesan-niclosamide in a final pH of pH 8.31 TBSS, where its day 2 value was 11.3 µM.

#### pH dependence and a lower solubility for the high-pH red crystals

**Fig. 17:**
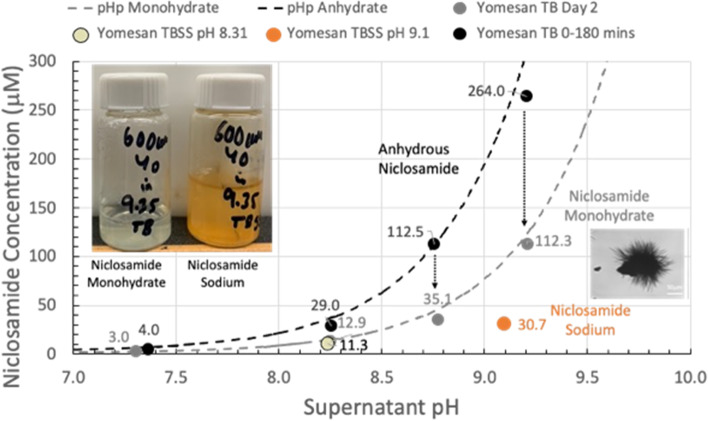
Supernatant niclosamide (µM) vs. supernatant pH: Expanded view of plot in Fig. 10 with added data for the TBSS system at final pHs of 8.31 (pale yellow-green filled circle, 11.3 µM) and 9.21 (orange-red-filled circle, 30.7 µM) as probably niclosamide sodium. Inset: photographic image of equilibrated samples showing the pale yellow-green appearance of the Yomesan niclosamide in nominal pH 9.35 TB (left) and the orange-red appearance of the Yomesan-niclosamide in nominal 9.35 TBSS (right). Also (lower right), microscopic image of the morphology of the grown crystals

For context, and to emphasize the differences, these two data points are plotted in Fig. 17 which is an expanded view of the pH dependence graph shown earlier in Fig. 10. A photographic image of the two vials of the equilibrated samples is also given as an inset, showing the pale yellow-green appearance of the Yomesan (Yo)-niclosamide in nominal pH 9.35 TB (left) and the orange-red appearance of the Yomesan niclosamide in nominal 9.35 TBSS (right).

Both were made simply by equilibrating excess Yomesan-niclosamide powder with the Tris buffer and Tris-buffered salt solutions. The data point for final pH of 9.21 TBSS is shown as an orange-red filled circle, and a final pH 8.21 TBSS is a pale yellow-green filled circle.

Interestingly then, in TBSS at a final pH of 8.31, Yomesan-niclosamide equilibrated to a supernatant concentration of 11.3 µM, which is similar to the equilibrated value in just Tris buffer (TB) of 12.9 µM (and similar yellow green suspension color). In contrast, at the higher final pH of 9.21, niclosamide achieved a concentration of just 30.7 µM, which is much lower compared to even the monohydrate, reflecting an even lower solubility for this niclosamide-sodium compound. What is most striking is that the excess undissolved material turns a bright orange-red color. As reported by Grifasi et al. (2015), orange-red crystals are associated with cocrystals of niclosamide-niclosamide sodium formed by kneading or ball milling of the dry powders. Here though these red crystals were formed simply by incubating excess, undissolved powdered Yomesan-niclosamide with a salt buffered solution. This new solid form could therefore be the co-crystal or more likely the sodium salt, niclosamide sodium.

**Fig. 18:**
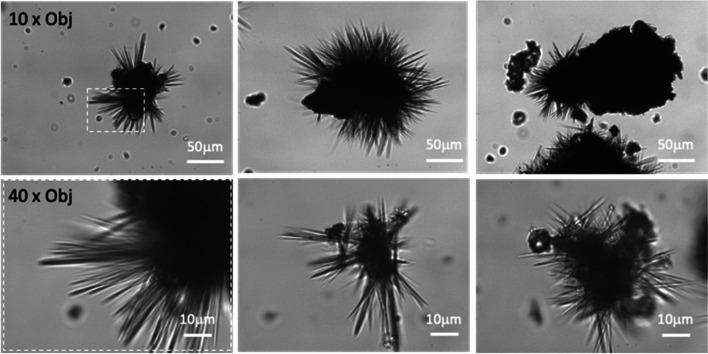
Optical microscope images of particles from the stirred sample undergoing dissolution and morphologic change at 25–30 min after addition in pH 9.33 TBSS. (Top) images with the 10× objective, scale bar is 50 µm; (bottom) images with the 40× objective, scale bar is 10 μm. In the first (top) image, the white dotted region-of-interest at 10× is shown magnified in the 40× (bottom) image. Bright field optics, Kohler illumination

To gain further insight into what was happening in the TBSS, the optical microscope was again used to look at samples at a time just after the peak in Fig. 16 of 98.5 µM, as the concentration was reducing. Figure 18 shows images of several different representative particles from the stirred sample undergoing dissolution and morphologic change, for a sample of the suspension at 25–30 min after addition in pH 9.35 TBSS.

These 20–30 min particle aggregates of Yomesan niclosamide were already forming many needles, growing out of the surface of the aggregates. It was very clear that this rapid needle formation was responsible for the decrease in supernatant niclosamide concentration, as it equilibrated with, what now seems to be, a much lower solubility polymorph of niclosamide-sodium or a niclosamide- niclosamide/sodium cocrystal.

**Fig. 19:**
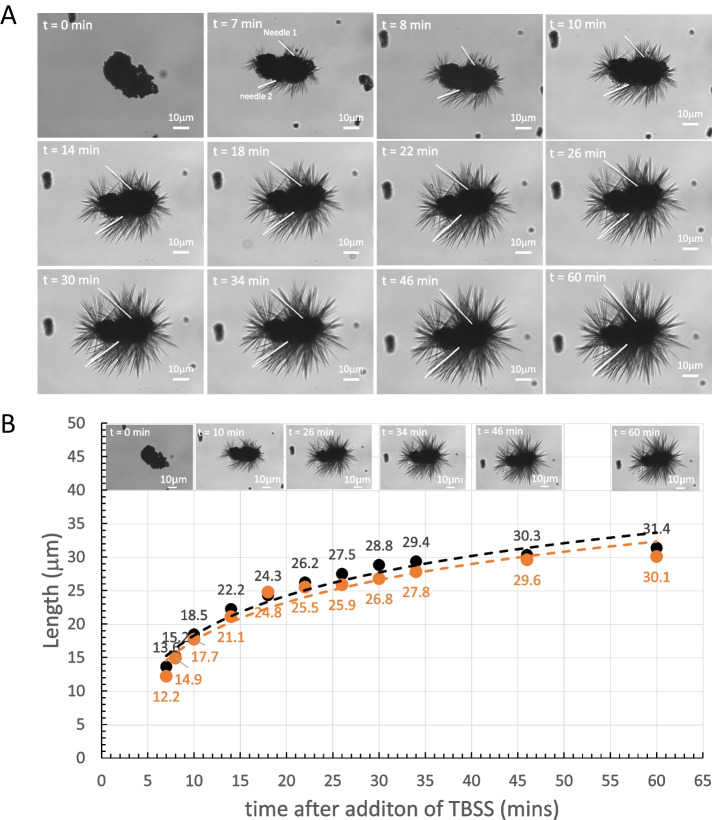
**A** Yomesan in pH 9.34 TBSS: Development of new needle growth vs time under static conditions (0–60 min). **B** Needle growth from Yomesan-niclosamide in pH 9.34 Tris-buffered saline: length (mm) vs time after addition of TBSS (min)

Finally in this section, as shown in Fig. 19, a few Yomesan niclosamide particles and a drop of TBSS were placed in a sealed microscope chamber (to limit any evaporation).

Observing over time, it was possible to see these needles grow and actually measure their growth rates. For a randomly selected particle-aggregate of niclosamide, Fig. 19A shows the development of new needle growth vs. time under static diffusion-controlled conditions (0–60 min). At *t* = 0 min, this particle aggregate showed the usual featureless particle aggregate morphology and little initial needle-like projections. By *t* = 7 min, needle growth had started and 2 of the needles were selected and followed over the next ~ 1 h of observation.

The growth of the needles was evaluated in Fig. 19B by considering the two needles highlighted, in white in Fig. 19A. Taking a calibration of 10 µm = 0.28 units on the image, needle lengths were measured by placing the white lines as best as could be judged by eye to match their origin and length for each time point image. The graph shows how the two needles grow to ~ 30 µm in length, at approximately the same rates and the data does fit a logarithmic linear growth of 15 to 18 µm per 1 h.

What these studies show is that including salt solution in the Tris buffer (to initially set a higher osmolality) only serves to convert the excess undissolved niclosamide to a new lower solubility salt-polymorph, but this only seems to occur at pHs well above its pKa. This may be associated therefore with the requirement of enough unprotonated negatively charged niclosamide to form the niclosamide sodium salt. These interesting preliminary observations of the formation of this purported red niclosamide sodium salt are now ready to be investigated in more detail using pure niclosamide and again motivate further dissolution, morphology, XRD, Raman, and DSC characterization on pure niclosamide itself.

### A scaled-up example: 125 mg/L of fresh Yomesan powder yields a 165 µM niclosamide solution

**Fig. 20:**
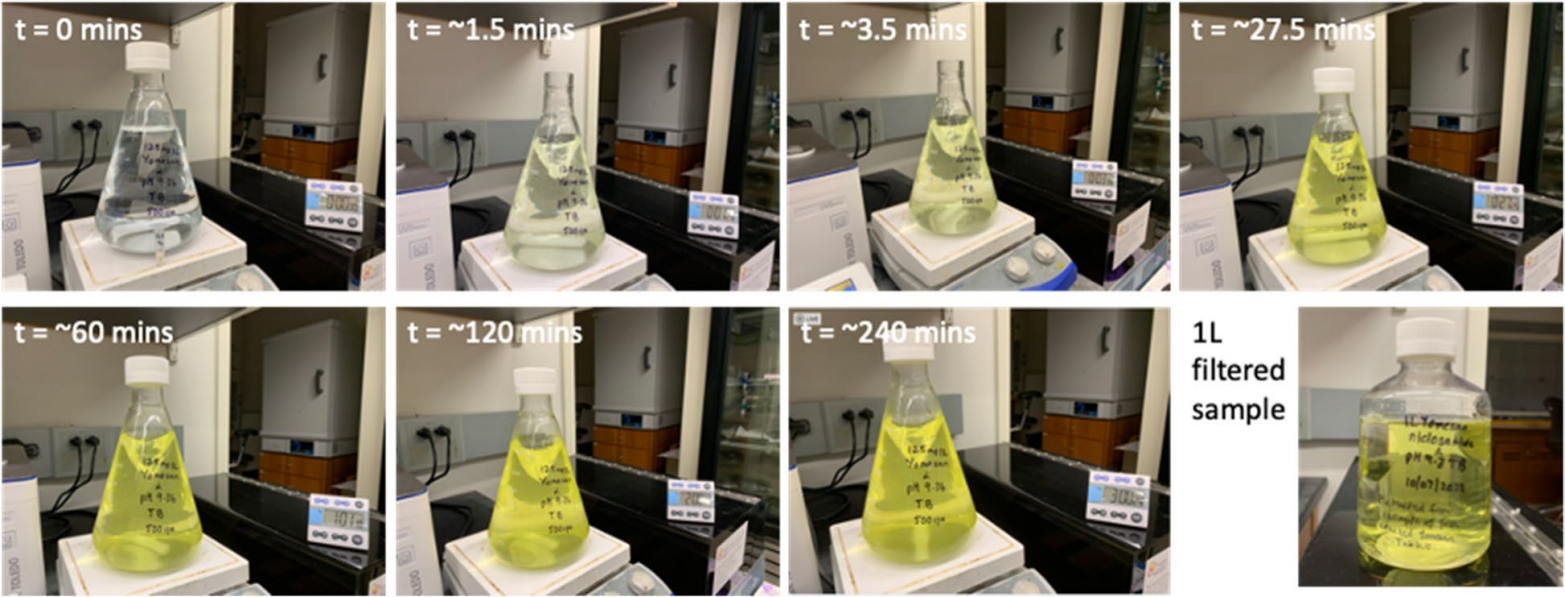
Dissolution of Yomesan-niclosamide at 1 L scale showing the increasing yellowness of the solution as the niclosamide dissolves over a period of 0–180 min. Far right, the filtered 1 L sample of 165 µM niclosamide in final pH 9.33

To finish off this report and provide a protocol for compounding pharmacies and other pharmacy-related labs to follow, as shown in Fig. 20, here is an example of making 1 L of niclosamide solution in Tris buffer by extracting it from the crushed Yomesan tablet material.

125 mg of fresh Yomesan powder was added to 1 L of nominal pH 9.36 Tris buffer in a conical flask stirred at 500 rpm with a magnetic stir bar. Already by* t* = 1.5 min, there is the characteristic yellow coloration of the supernatant. This yellowness increases with time as more and more niclosamide dissolved from the suspended and stirred Yomesan tablet material. By 60 min, the 1 L suspensions had a quite pronounced, yellow coloration that got a little more yellow out to 240 min (4 h).

As with the lower volumes, samples were taken at regular time points, filtered through a 0.22 µm filter. The clear supernatant was analyzed by UV-Vis to obtain the spectra and niclosamide concentration at 333 nm.

**Fig. 21:**
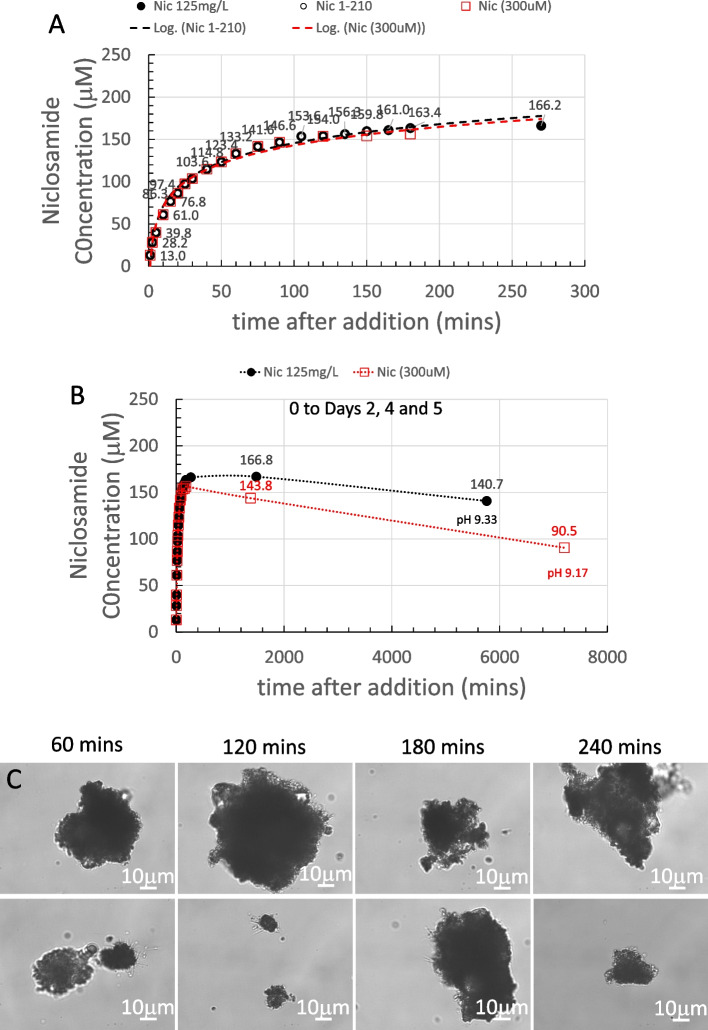
1 L scale up. **A** Dissolution profile for a 1 L sample of 125 mg/L fresh Yomesan, 0–180 min (black filled symbols), and a 300 µM 20 mL sample (red squares) from Fig. 4A. Dashed lines are the logarithmic fits to both sets of data. **B** Dissolution profile for both samples 0 to days 2, 4, and 5 at final pHs of 9.33 and 9.17, respectively. **C** Optical microscope representative images of niclosamide aggregates for the 1 L sample at 60 to 240 min of dissolution-stirring that show very little change in morphology.

In order to guage the time-point of relative maximum dissolution, the supernatant niclosamide concentration was actually monitored in real time and plotted throughout the dissolution. As shown in Fig. 21, the UV-Vis absorbance at 333 nm reached 133 µM at 60 min corresponding to the intense yellow coloration. It then reached a relatively constant value at 180 min when the niclosamide concentration was ~ 165 µM. At this point, 20 mL of the suspension was poured into a scintillation vial to continue the stirring and determine subsequent concentrations and excess particle morphologies. The rest of the one-liter suspension was vacuum filtered and collected as shown in the far-right image in Fig. 20.

As seen with all earlier samples at the lower 20 mL scale, for the 1 L sample, dissolution in Fig. 21A was logarithmic (white symbols) out to 180 min. Data in Fig. 21B is after an overnight stir for the 20 mL sample (24.75 h) on day 2. Stirring this fresh sample did not lose any concentration and maintained a supernatant niclosamide at 166.8 µM. Given that the original 125 mg of added Yomesan powder was equivalent to 300 µM niclosamide, this represents a 55% yield.

Thus, fresh Yomesan powder maintained a mostly anhydrous state as reflected in the representative microscope images of the particulate niclosamide aggregates in Fig. 21C that showed little, if any, needle morphology growth throughout the whole 240 min of dissolution-stirring on day 1. On very rare occasions, as shown in Fig. 21C at 60 min, there were only a few needles on a few particles. Thus, by not allowing any chance of humidity and storage, to create what is assumed to be precursor monohydrate, inhibits massive needle formation (as in Figs. 13 and 14) and preserves the solution concentration.

There was also hardly any change on day 2 suggesting that there was little conversion or growth of the lower solubility monohydrate for this freshly ground sample, and the amount of niclosamide in supernatant solution again remained high at 166.8 µM. When equilibrated for another 3 days standing at room temperature the sample did however show a decrease in supernatant niclosamide concentration to 140.7 µM (Fig. 21B). This was accompanied by a slight change in morphology including some needle-shaped crystals on the undissolved particulate aggregates (images not shown).

By comparison, the older sample from Fig. 4A (red squares) that was also at 300 µM equivalent niclosamide, but at the smaller 20 mL scale, did show in Fig. 21B some reduction in niclosamide concentration at the 23 h time point to 143.8 µM, and, at day 5 after standing, it was reduced to 90.7 µM for the supernatant niclosamide. The Yomesan sample used for this earlier experiment was from a different crushed Yomesan tablet that had nevertheless been maintained mostly under vacuum. Of course, if the niclosamide used in the original manufacture of the tablets was not tightly checked or controlled for in terms of anhydrous and, especially, monohydrate content, and there is a mixture of the two, then there could be some conversion upon dissolution-stirring.

The final pHs were also slightly different, pH 9.33 vs. pH 9.17. Since there is a pH dependence to niclosamide’s total solubility (Needham 2022), and at these higher pHs the curve is quite steep, it would be expected that the amount of niclosamide in solution would reflect any difference in final pH. This final pH is mostly controlled by the 20 mM Tris buffer, but the weak acid niclosamide can change that; the buffer could be made more concentrated to, say, 100 mM, which would also serve to increase the base osmolality to be closer to 290 mOsm physiologic without using sodium chloride. In any event, this simple dissolution protocol scaled up to a 1 L volume successfully extracts ~ 165 µM niclosamide from crushed Yomesan tablets into nominal pH 9.35 Tris buffer in ~ 3 h. While scientifically interesting, with regard to establishing an effective protocol for extraction of niclosamide from the approved tablets, longer stirring times are just something to avoid in order to obtain the maximum amount of niclosamide in supernatant solution.

## Discussion

### Niclosamide can be readily extracted from crushed Yomesan tablets into simple pH buffer

The main point of these studies was to understand the processes occurring when crushed Yomesan tablets are dissolved in Tris buffer. Based on the findings carried out at a small 20 mL scale, it was then to establish an effective protocol for extracting niclosamide at the 1 L scale, as a basis for creating new, more readily approvable, oral, and nasal sprays against COVID-19 and other respiratory infections. As shown by all the 20 mL data as a function of concentration and pH and demonstrated at the 1 L scale in Fig. 20, and quantified in Fig. 21, this was achieved. After 3 h of dissolution of 125 mg/L Yomesan, which is 100 mg/L niclosamide equivalent (i.e., just one fifth of a 500 mg Yomesan-niclosamide tablet), filtration of the suspension to remove all excess niclosamide and other excipient particles, a 1 L solution of 166 µM niclosamide at pH 9.3 was obtained.

The oral cavity can easily withstand a modest pH 9.5 (green tea is pH 9.0 to 9.5 and so is alkaline drinking water) and so, this solution could be used as is, as an early treatment oral spray for a sore throat and to limit person-to-person spread. To make the nasal spray preventative that necessitates frequent use, the pH of the niclosamide solution can be reduced to a nasally safe pH 8.3 and the solution appropriately diluted with Tris buffer to 20 µM where niclosamide is still soluble.

### The low therapeutic concentration needed provides massive opportunities for simple scale-up manufacturing

As we have shown in recent cell culture studies in the most relevant nasal and bronchial epithelial cells, niclosamide is absolutely safe at niclosamide concentrations less than 100 µM (unpublished results). The 100 µM dose that is lethal to the HNE and HBE cells was actually measured after a 24 h exposure to niclosamide solutions at 37 °C. It is unlikely that a sprayed intra-nasal dose would expose the cells for 24 h. Thus, this 20 µM niclosamide concentration is 20 times the efficacious inhibitory IC_100_ concentration (measured to be 1 - 2 μM in vero6 and other cells) needed to stop nasal and bronchial cell infection yet is safe to those cells.

To put all this into a mass-volume-perspective, just one fifth of a Yomesan tablet yields 1 L of 165 µM niclosamide solution at a nominal pH 9.35. Adjusting the pH to 8.3 and diluting to a 20 µM niclosamide solution would yield 8.25 L, and so, 41.25 L per single tablet. One 4-tablet pack of Yomesan could therefore readily make 165 L of the 20 µM solution. When used at 10 mL per nasal spray bottle, this gives 16,500 bottles from one Yomesan 4-pack. One spray dose is 100 µL of 20 µM niclosamide and so, there would be 100 doses per bottle at just 0.65 µg of niclosamide per spray. One can therefore easily imagine generating one million bottles from just 60 packs of Yomesan. This would provide 100 million single spray doses from such a simple dissolution protocol for distribution and use throughout the world.

### Regulatory approval?

#### Do we really need to start from square one with a 505(b)(2)?

While it is clearly possible to start with a niclosamide supplier of pharmaceutical grade niclosamide and go through the whole 505(b) (2) regulatory process (REF: https://www.fda.gov/regulatory-information/search-fda-guidance-documents/applications-covered-section-505b2) with an appropriate 505(b)(2) partner, this is involved, costly, and time consuming. Also, while the USA has all but “returned to normal”, there is still some urgency in the face of new increases in cases in Europe for this one virus (WHO 2022), and potentially more to come (Marani et al. 2021). Also, other respiratory infections that niclosamide has shown broad spectrum activity against (Xu et al. 2020) and could use a simple preventative and early treatment option. This is why it is proposed that such a simple adjustment in administration of an existing, approved, and commercially available dosage form could make this niclosamide solution-preventative and early treatment spray more widely available, while not requiring starting from square-one with the FDA and other regulatory bodies.

#### Could favorably impact the elderly, other immune compromised and those with limited access to vaccines and anti-virals

As such, this could be especially important for the elderly where, unconscionably, 83.5% of COVID deaths were aged over 60 year. Yet they only make up 21.9% of the whole population (Health_Equity_Tracker 2022). And for people over 50, there were almost 1 million deaths (973,896) reported by Sept. 2022 (Elflein 2022). Also at high risk are the immune compromised or people with weakened immune systems due to recent surgeries, age, genetics, having a chronic illness, or by taking certain medications such as for cancer. While the majority have gone back to business as usual, such people are more likely to (still) become severely ill with COVID-19—the COVID-19 pandemic rages on for people who are immunocompromised (Rubin 2022). There is Evusheld, that is currently paid for by the US government and so is ~$10 for 3 mL (Recht 2022), but according to White House officials, the USA will likely run out of Evusheld by the end of the year (THE_WHITE_HOUSE 2022). In any event, as warned by the FDA, it is becoming less effective the more the virus mutates (FDA 2022).

Looking beyond the USA, there are still regions of the world that do not have easy- or widespread-access to vaccines and other medications for COVID-19 and other respiratory viral infections. For example, according to the WHO, as of 10 July 2022, 282 million people on the African continent had completed their primary series, representing (only) 21.1% of Africa’s population.

Given that the tablets are already approved and available (and Bayer could expand manufacturing and distribution; they have done it before (Bayer 2019)), compounding or other pharmacies could simply prepare the solutions in-house by following the methods and recipes reported here and provide them locally. This kind of simple solution preparation may even, eventually, attract the attention of the niclosamide tablet producers, including Bayer, Germany (the original producer of Yomesan), and other human-prescribed generics, like*Niclosig*, by HAB Pharma, India, and even livestock versions like *Luxiaoliunpian* (Hanzhong Tianyuan Pharmaceuticals, China) to make availability and distribution more accessible (or at least that’s the hope). Similar dissolution data is available on these generics from the author.

Normally, for intra nasal administration, it is expected that a 505(b)(2) application would have to be made. Yomesan already contains pharmaceutical grade niclosamide, and the concentrations (20 µM) and 0.65 mg per sprayed dose reported here are ultralow. Also, other much more concentrated (25 mM) and more toxic (DHSS 2002; Chemicals 2020) niclosamide ethanolamine nasal sprays are currently being trialed in humans (Union-Therapeutics 2021, 2022). Thus, once this simple extraction procedure becomes known, perhaps expedited approval could be obtained, especially in the face of an on-going pandemic that is escaping current antibody treatments (Axe 2022) and requires multiple vaccinations and boosters to maintain an otherwise fading immunity (Naaber et al. 2021).

While vaccines and natural immunity need regular updates (Hachmann et al. 2022), a nasal spray would last a lifetime. And then there is the triple-demic targeting children (Fischer and Regan 2022), where niclosamide has already shown activity in cell infection models for RSV (Niyomdecha et al. 2021) and Influenza (Jurgeit et al. 2012).

#### It just needs testing

Cell studies are overwhelmingly positive that niclosamide at very low concentrations (depending on host cell type) can stop viral infection. The extent to which oral and nasal sprays can provide short-term protection against any virus exposure or early treatment options that also limit spread still needs testing. The hope is that the data in this paper and subsequent studies that further optimize the formulation to add an additional non-mucin penetrating depot of niclosamide that can nevertheless dissolve, perhaps combined with mucin-binding polymers that have been shown to also limit viral entry into cells (Bentley and Stanton 2021), will attract the right partners (governments, infectious disease institutes, and even companies) to take this forward and provide a preventative and early treatment option throughout the world.

## Summary and conclusions

The data presented here show that, indeed, niclosamide can be readily dissolved and extracted from the tablets as crushed powders, and yield tens of liters of buffered niclosamide solution from just one 500 mg tablet. While this process appears initially simple and straightforward, there were underlying principles that became important to consider. Interestingly, the amount of niclosamide obtained in the supernatant during the dissolution process, and especially when at equilibrium, depended critically on the nature of the initial polymorph (or mixture of polymorphs) exhibited by the niclosamide used in the manufacture of Yomesan (and in the other two different tablets studied, Niclosig and Luxiaoliunpian).

As expected from previous studies on pure niclosamide (Needham 2022), all niclosamide samples showed a dependence for the supernatant niclosamide concentration on buffered solution pH, the time of equilibration, and interestingly the presence of sodium (as sodium chloride). The final equilibrium concentration of niclosamide extracted was particularly sensitive to the presence and conversion of the excess powdered tablet niclosamide to one or more polymorphs, i.e., whether or not it was either purely anhydrous, or additionally contained a mixture of anhydrous, plus one or more of the monohydrates that are reported to have a lower aqueous solubility (van Tonder et al. 2004). These studies on the tableted niclosamide and its complex morphologic and solubility behavior now motivate a more in-depth study of niclosamide per se involving additional analytical techniques such as Raman, XRD, DSC and TG.

One of the main goals of this study on niclosamide dissolution was to lay the foundation for pursuing the idea that niclosamide could be simply extracted into supernatant solution from already approved niclosamide tablets. The thinking was that, if this could be easily accomplished, then a niclosamide-based oral-throat spray and then a nasal spray preventative could be readily available. As recommended by Bayer (2008) and previously approved by the FDA (1996), “The pleasant tasting tablets should be thoroughly chewed to a fine paste and washed down with a little water”, and “For young children, the chewable tablets should be crushed to a fine paste and given with a little water”. Thus, a much more diluted oral spray could be more easily approved than starting from commercial niclosamide and building a 505(b)(2) formulation from scratch. Instead of using 10 mL of tap water to make the 500 mg tablet into a paste for children to take, just one fifth of the tablet can be dissolved in 1 L of Tris buffer to yield a 165 µM solution, and excess undissolved material is filtered out.

Thus, the results reported here are intended to provide a guide as to how to utilize, especially, commercially available, and approved tablets of already pharmaceutical grade niclosamide, to generate simple aqueous niclosamide solutions that could, in principle, be used to mitigate a host of respiratory infections as a preventative nasal spray and as an early treatment oral-throat spray. This could be done throughout the world to provide local niclosamide solutions, administered in the same manner as oral tablets but as much more dilute aqueous sprays for early treatment oral-throat sprays and, when tested, preventative nasal sprays.

## Supplementary Information

**Additional file 1:** Supplementary Information.

## Data Availability

The datasets generated during and/or analyzed during the current study are available from the corresponding author on reasonable request.

## Footnotes

1 Except in the USA where Bayer withdrew its regulatory approval in the mid-1990s (FDA 1996) and so is only available upon request from the CDC.
