# Supplementary material for: Extraction of niclosamide from commercial approved tablets into aqueous buffered solution creates potentially approvable oral and nasal sprays against COVID-19 and other respiratory infections
Source: AAPS Open. 2023 Apr 14;9(1):9. doi: 10.1186/s41120-023-00072-x (PMC10101733; doi:10.1186/s41120-023-00072-x)
Supplement: Supplementary file 1 — Additional file 1: Supplementary Information. [file 41120_2023_72_MOESM1_ESM.pdf]

Supplemental Information

Extraction of Niclosamide from Commercial Approved Tablets into Aqueous Buffered Solution Creates Potentially Approvable Oral and Nasal Sprays Against COVID19 and Other Respiratory Infections

David Needham[1, 2]
d.needham@duke.edu

[1] Department of Mechanical Engineering
and Material Science, Duke University, Durham,
North Carolina 27708, USA

[2] Professor of Translational Therapeutics,
School of Pharmacy, University of Nottingham,
Nottingham NG7 2RD, UK

KEY WORDS. niclosamide · dissolution · Yomesan · Luxiaoliunpian · Niclosig · pH buffer · universal nasal-throat-spray · COVID19 · respiratory viral infections

Table of Contents

Supplemental Information 3

S1. Instrument Resolution (Photometric accuracy) 4

S2. Vanillin 6

S3. Talc and Cornstarch 8

S4. Fraction of Niclosamide Extracted from Yomesan Powder 9

S5. Initial Rates of Dissolution 12

S6. Niclosamide in Tris Buffered Saline Precipitates or Grows "Wheatsheaf" Crystal Morphologies 14

S7. Comparative UV-Vis Spectra 15

S7.1 Concentration Dependence *15*

300μM Fig. S8A 15

600μM Fig. S8B 15

1mM Fig. S8C 15

S7.2 pH Dependence *17*

pH 7.41 Fig S9A 17

pH 8.35 Fig S9B 17

pH 8.85 Fig S9C 17

pH 9.35 Fig S9B 17

S8. Comparative Dosing Between Yomesan and an Oral or Nasal Niclosamide Spray 19

References Cited 19

Supplemental Information

Supplemental information provides added and underlying data for the main text. It includes:

- UV-Vis absorbance measurements that conformed the instrument resolution (Photometric accuracy)
- UV-Vis absorbance spectra of pure vanillin as a function of its concentration and pH of Tris buffer that identify it as the absorbing "impurity" in the Yomesan powder
- UV-Vis absorbance spectra Talc and Cornstarch that show they did not affect the results
- The measured peak maximum niclosamide concentrations and % niclosamide that were extracted by dissolution of Yomesan tablet powder in nominal pH 9.35 Tris buffer
- The initial rates of dissolution data in **Figs. 4** and **5** (main text) are quantified as µM niclosamide dissolved per min of dissolution vs the initial Yomesan niclosamide equivalent concentrations added.
- The way in which niclosamide precipitated into in Tris Buffered Saline Solution (TBSS) forms a "Wheatsheaf" morphology
- Comparative UV-Vis Spectra for dissolution that support the plots in the main text for the:
  - Concentration Dependence
    - 300µM
    - 600µM
    - 1mM
  - pH Dependence
    - pH 7.41
    - pH 8.35
    - pH 8.85
    - pH 9.35
- Comparative dosing between Yomesan and an oral or nasal niclosamide spray is also included because, if used as an oral spray, it would essentially be applied locally to the same buccal and throat epithelium as the "thoroughly chewed" 2 grams of Yomesan tablets. The amount per 100uL sprayed dose though would be 5.4 micrograms for a 165µM niclosamide oral dose, and only 0.65 micrograms for an intranasal 20µM niclosamide solution.

S1. Instrument Resolution (Photometric accuracy)

An expected solubility for niclosamide of only ~2μM in the low pH range corresponds to an absorbance of only 0.02 Absorbance units (AU). This is only an order of magnitude higher than the resolution (photometric accuracy) of the UV5nano which is quoted as +/- 0.005 Absorbance units. Note: in the manual photometric accuracy is quoted < ± 0.01 A (potassium dichromate, Ph.Eur./USP method).

It was also necessary to evaluate this resolution to make initial measurements of supernatant niclosamide concentration early on in the dissolution process of the crushed tablet powder. Resolution therefore was tested with nine samples in the same quartz cuvette (used in the same direction) which was washed and refilled for each series of three measurements

As shown in **Fig. S1A**, (next page) are the Raw Spectra for the Blanks containing filtered Tris Buffered Saline Solution (TBSS) at pH 9.34. Three sets of blank spectra were taken (three consecutive measurements for each).

Plotted in **Fig. S1B** is the average relative absorbance versus sample number. These are the averages of all 2,050 data points from 290nm to 700nm at 0.2nm per point. The first series shows three values spread from negative to positive, at Relative Absorbance Units (AU) of -0.00219,

00144 and 0.00375 The second series shows all three give negative values: two, close together and at the 0.005 limit of -0.00491 and -0.00552, and then close to the zero at -0.00033. The third series also shows increasing average with sample test number of -0.00472, and then positive, 0.00115 and 0.00199.

The average Absorbance for all 9 scans was -0.00104 +/- 0.00342 AU, while the average lower and upper average values were -0.00504 and 0.00503. It was therefore confirmed that the instrument and experimental technique showed the expected range for the photometric accuracy of ~ +/- 0.005 AU.

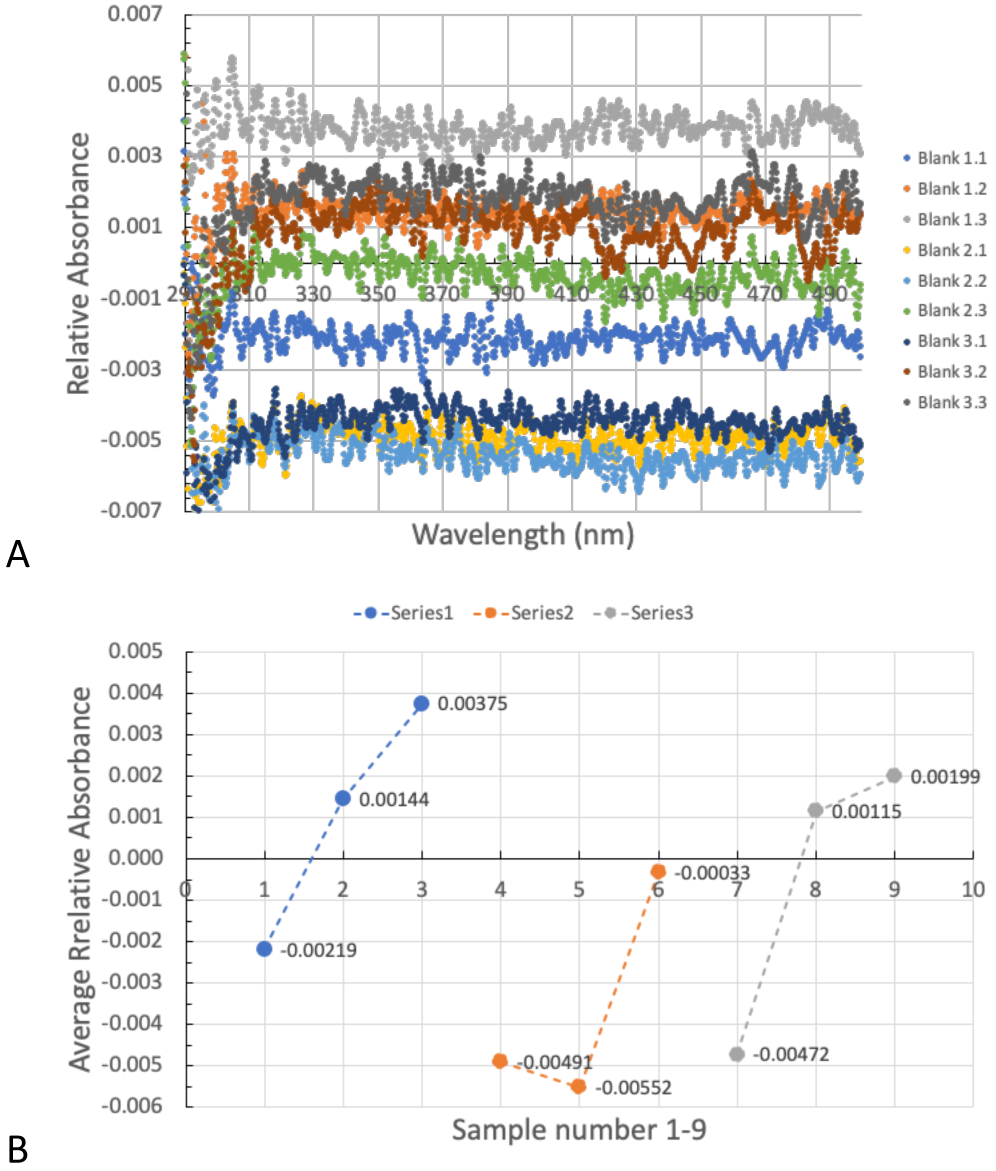

Fig. S1 Photometric Accuracy. **A)** Raw Spectra for Blanks. Shown are three sets of blank spectra (three consecutive measurements for each) made for filtered Tris Buffered Saline Solution (TBSS) at pH 9.34. **B)** Average Relative Absorbance vs Sample number of 3 scans for each of 3 samples as averages of all 2,050 data points per spectrum from 290nm to 700.

S2. Vanillin

In order to prove the material was vanillin an interesting side study was completed. 100µM vanillin solutions were made up in pH 8.35 and 9.35 Tris buffer and serially diluted to provide calibration spectra for comparison with the Yomesan spectra. As shown in **Fig. S1A** and **B**, the UV-Vis spectra of vanillin at pH 8.35 and 9.35 in Tris buffer have a single peak at ~347nm. The spectra and peak agreed well with the experiments of Shu et al [1], and their test spectra, shown in **Fig S1D**, of vanillin in 10mM sodium borate at pH 8.0, and an absorption maximum at λ_{max} of 347 nm. Interestingly, at pH 7.0, as shown in **Fig. S2**, the vanillin spectrum showed two absorbance peaks at 316nm and 347nm, consistent with that reported by others including NIST [2] (pH was not given but assumed to be water). This side study on vanillin was important because it identified the "impurity" in the Yomesan spectrum as vanillin and has actually provided confirming data, newly presented together with vanillin's own pH-dependence, and the spectra that can be subtracted from the Yomesan dissolution spectra to reveal the niclosamide in supernatant solution.

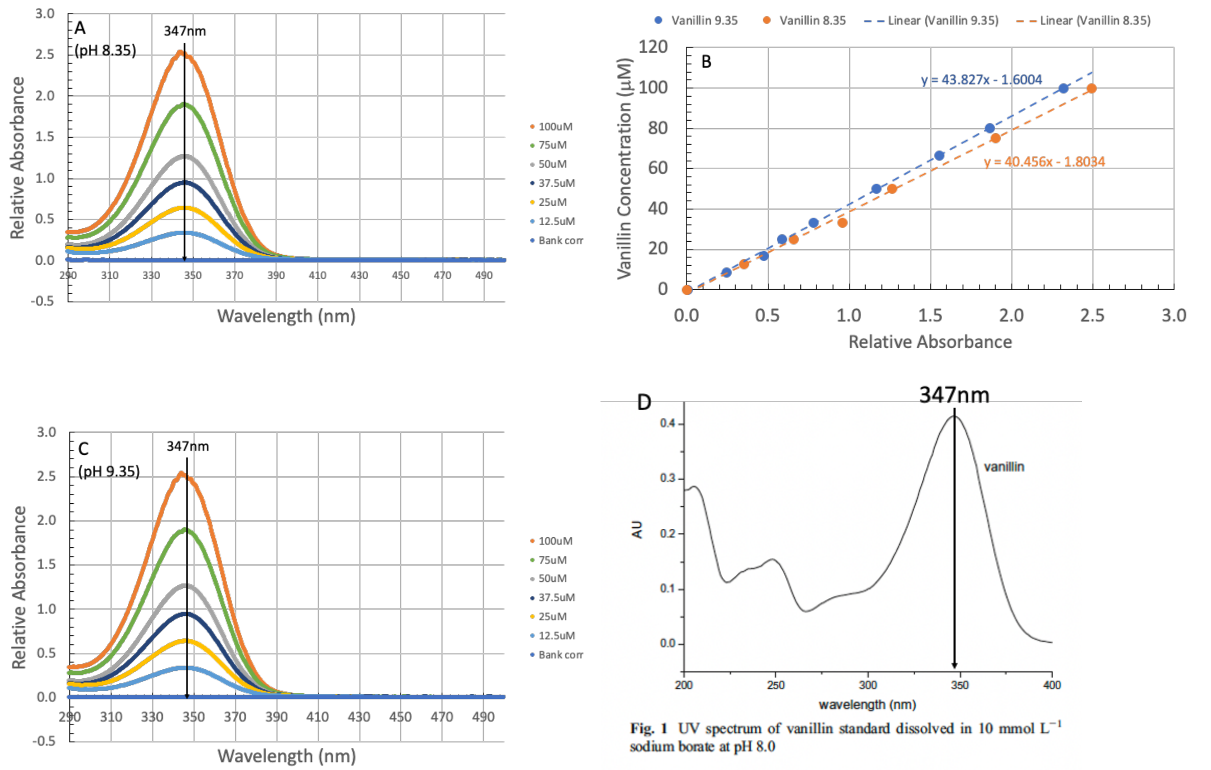

Fig S2. UV Vis spectra of vanillin. UV-Vis spectra (relative absorbance versus wavelength (nm) for serial dilutions of 100µM vanillin in **A)** pH 8.35 and **B)** pH 9.35 showing the λ_{max} of 347 nm; **C)** calibration plots of relative absorbance versus vanillin concentration (µM) for pH 8.35 and 9.35; **D)** UV spectrum of vanillin standard dissolved in 10 mM sodium borate at pH 8.0 by Shu et al, [1] agrees with the presented spectra and λ_{max} of 347 nm.

Plotting the absorbance of the 347nm peak versus concentration in **Fig. S2C**, shows that these two separate samples, made up in two different buffer solutions, one at pH 9.35 and the other at pH

8.35 give a linear (calibration) relationship and agree fairly well, with slopes of 40.8μM/AU and 40.5 μM/AU.

For completion, because Yomesan was also tested at ~pH 7, the impurity spectrum would also need to be accounted for there too. As shown in **Fig. S3A**, vanillin at pH 7.06 actually has a completely different serial dilution spectrum to the higher pH spectra. The spectra at pH 7.06 has two absorbance peaks at 316nm and 347nm and are consistent with those reported by others including NIST [2] (pH is not given but assumed to be water).

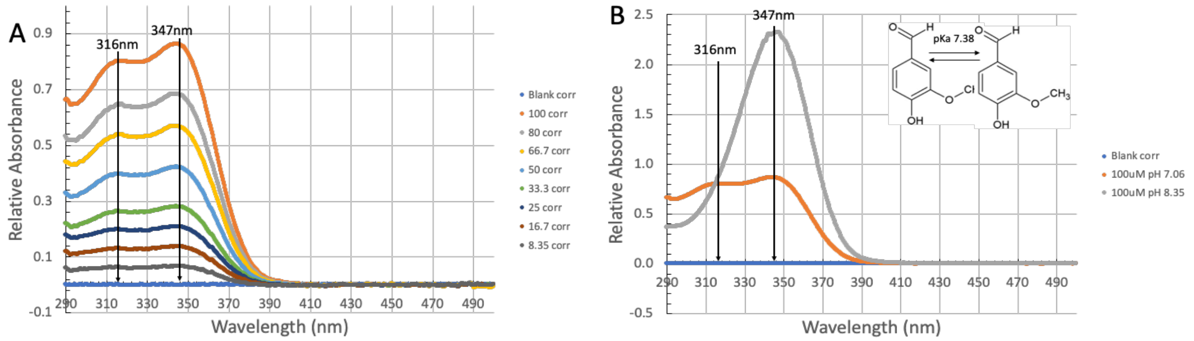

Fig S3. Vanillin at pH 7.06 below its pKa (of 7.38). **A)** UV-Vis spectra of Vanillin at pH 7.06 showing two absorbance peaks at 316nm and 347nm. **B)** Comparison between UV-Vis spectra at pH 7.06 and 8.35 for the two species, -- the protonated acid and deprotonated salt.

Interestingly, then, like niclosamide, vanillin is also a weak acid. As reported by Shu et al, [1] the pKa value of vanillin is 7.38, which is actually quite close to that of niclosamide of 7.12 as derived earlier for pH dependent solubility data [3]. As also shown in **Fig S3B**, at a pH of 8.35, that is above vanillin's pKa of 7.38, the 317nm peak all but disappears as the more dominant, presumably, salt peak at 347 increases with increasing domination of the negatively charged salt.

Thus, when the pH is >7.38, vanillin in solution is increasingly negatively charged and as dictated by weak acid equilibria and the Henderson Hasselbalch equation, will have a higher total solubility than when the pH is <7.38, where it is protonated, in a neutral state and less soluble.

While the pHs were not specified, the solubility of vanillin has been reported to be 67.65mM [4], 72.3mM (Yalkowski, [5]) and calculated, at pH 7.06, by MarvinSketch (Chem axon) to be 129mM. Thus, at these concentrations of 100uM and less, both the weak acid and the deprotonated salt are expected to be water soluble.

This side study on vanillin was important because it identified the "impurity" in the Yomesan spectrum as vanillin and has actually provided confirming data, newly presented together of vanillin's own pH dependence, and the spectra that can be subtracted from the Yomesan dissolution spectra to reveal the niclosamide in supernatant solution.

S3. Talc and Cornstarch

For completion, shown in **Fig. S4** are the spectra of saturated solutions of talc and cornstarch in pH 7 tris buffer. given that common excipients used as binders and disintegrants in oral tablets include talc (USP Medisca) and cornstarch (supplier), in all tablets. Although not listed on the packaging, noting that the tablet weight of the 500mg niclosamide was almost doubled by the excipients, and that and talc was especially evident in optical microscope images of the Niclosig, spectra of saturated solutions of talc and cornstarch were obtained, as shown here.

Both talc (290nm – 410nm) and cornstarch (290nm – 400nm) do appear to absorb in the same range as niclosamide. However, as can be appreciated, even the saturated solutions of these USP excipients, give a very low averaged absorbances, ~0.004 and 0.005 respectively in the same range as the resolution of the spectrometer (0.005A).

Nevertheless, given the apparently high talc amount that would provide a saturated solution, the talc spectra were subtracted from the Niclosig absorbances when made in pH 7.0 Tris Buffer.

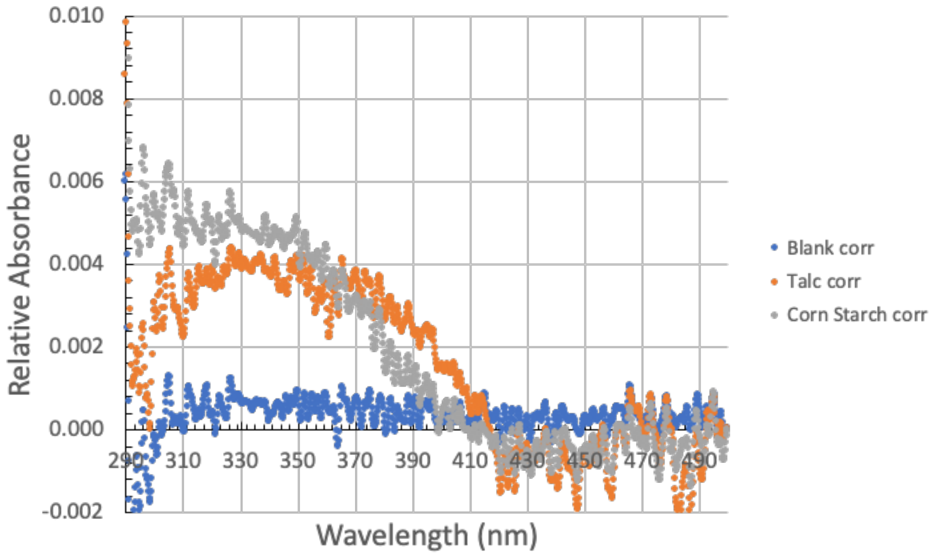

Fig. S4 UV-Vis Spectra for Talc and Corn Starch (saturated solutions in pH 7 Tris Buffer, same Quartz cuvette, 3-Averaged, Baseline-Corrected)

S4. Fraction of Niclosamide Extracted from Yomesan Powder

While it is expected that niclosamide of a given polymorph would have a consistent concentration at a given pH, (as reported earlier), the supernatant niclosamide concentration extracted from the tablet material may not necessarily reach, or fully represent, that concentration. For completion and to give the numbers as to what might be expected in a scaled-up extraction, **Fig. S5A** and **B** show the measured peak maximum niclosamide concentrations and % niclosamide that were extracted by dissolution of Yomesan tablet powder in nominal pH 9.35 Tris buffer.

As can be seen in **Fig. S5A** none of the samples reached the expected value measured for the anhydrous AK Sci niclosamide in the previous publication, [3], which at pH 9.22 was 344μM. This could reflect some drug binding within the tablet excipient materials (cornstarch, talc, …), as well as the conversion to the lower solubility polymorph.

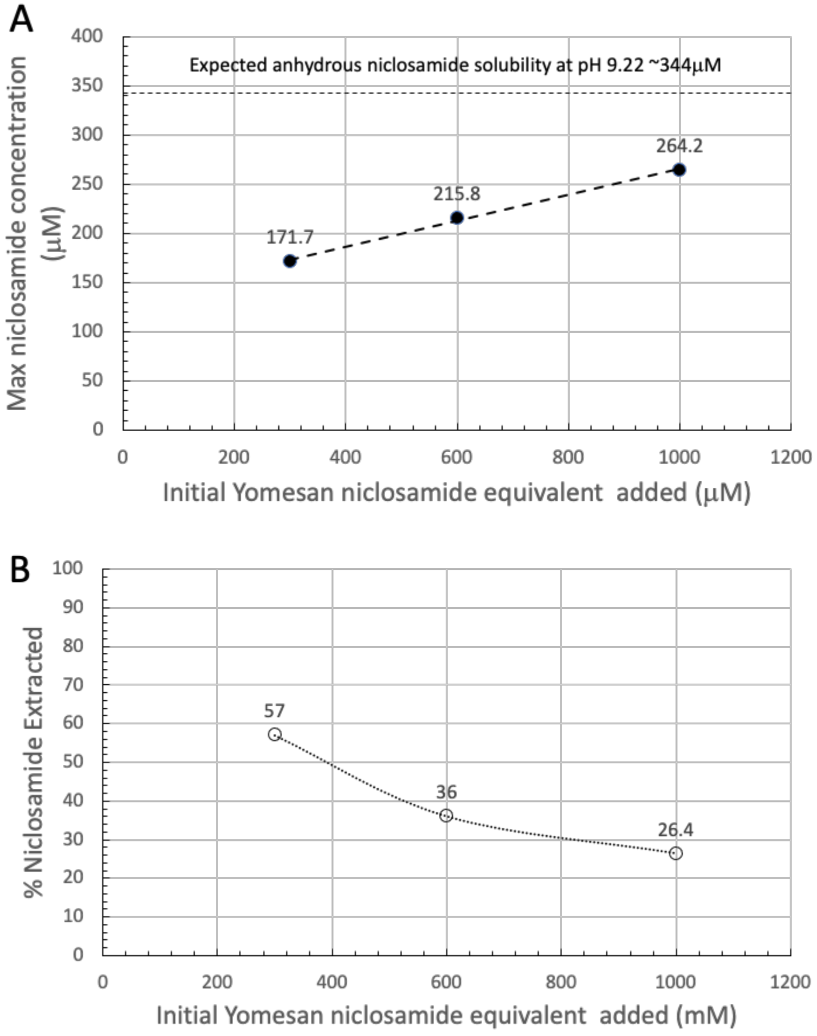

Fig. S5. A) Maximum supernatant niclosamide concentration and **B)** % niclosamide extracted by dissolution of Yomesan tablet powder in nominal pH 9.35 Tris buffer versus initial amount of Yomesan niclosamide equivalent powder added (µM) for the 300µM, 600µM and 1mM (achieved at 3 hrs,1hr, and 1 hr respectively).

As discussed in the main text, in association with the morphological evidence by optical microscopy, it seems clear that niclosamide used in the tablets is a mixture of anhydrous and monohydrate, and it is the conversion or growth of monohydrate crystals on the anhydrous aggregates that results in the lowering of the supernatant concentration.

Thus, taking these three concentration results together, as shown by the black filled symbols, in **Fig. S5A**, the extraction of niclosamide from Yomesan tablet powder increases, as might be expected with increasing amounts of niclosamide added. However, even for 1mM niclosamide equivalent (~3x excess material) it does not achieve the expected pH-dependent concentration of 344µM that a pure niclosamide sample does of an anhydrous niclosamide (AK Sci) at the same pH of 9.22, as measured earlier.

In **Fig. S5B**, the same data is plotted now as a percent of niclosamide that is extracted from the given amount of added Yomesan powder, as an equivalent total niclosamide concentration. As the graph shows, increasing the amount of available niclosamide in the added suspended powder does not proportionately increase the amount of niclosamide that can be extracted; in fact, the extracted yield% actually decreases and so behaves with diminishing returns.

What this data shows then is that niclosamide can certainly be extracted from Yomesan tablets into nominally pH 9.35 Tris buffer achieving concentrations in the 170μM to 260μM range.

However, with a faster rate of dissolution for the greater amounts available (larger surface area of more material) and therefore increased concentration in supernatant solution, it appears that the excess material initiates the growth of the lower solubility polymorph that starts to bring down the overall amount of niclosamide in supernatant solution. Thus, to reiterate, some niclosamide either remains bound to one or more excipients, and/or the growth of the lower solubility monohydrate polymorphs depletes the supernatant niclosamide concentration.

S5. Initial Rates of Dissolution

It is also clear from the solid straight lines in **Figs. 4** and **5** (main text) that the initial rates of dissolution increase as more powder is available, i.e., 1mM Yomesan niclosamide dissolves the fastest and the 300µM dissolves the slowest. This is quantified in the next two graphs in **Fig. S6**. Measurements of supernatant niclosamide concentration for the initial dissolution of Yomesan niclosamide over the first few minutes give the initial dissolution rates, as shown in **Fig. S6A**.

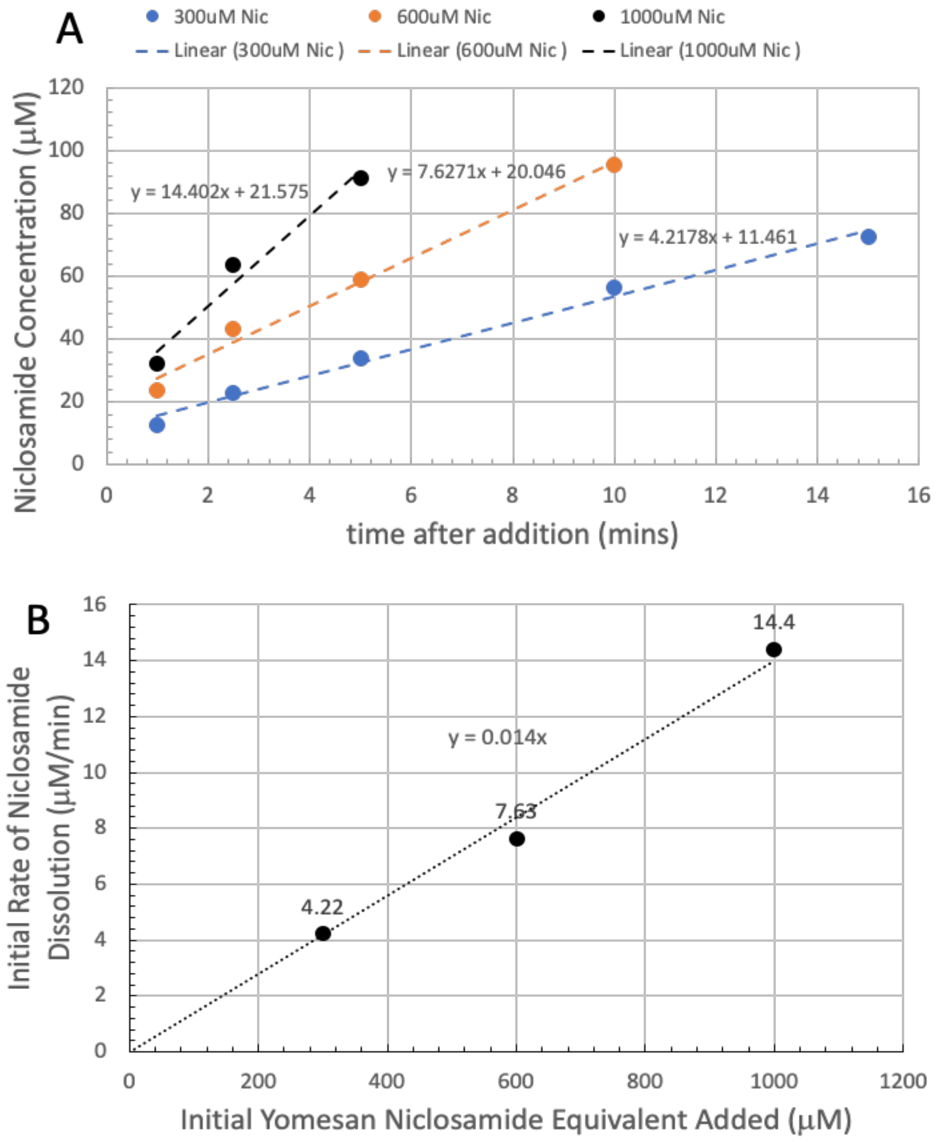

Fig. S6. Initial rates of Yomesan-niclosamide dissolution. **A)** Niclosamide concentration (mM) versus time after addition for the first few minutes from **Fig 5** of the powder dissolution experiment. **B)** Initial rates of Yomesan-niclosamide dissolution (µM/min) vs initial Yomesan niclosamide equivalent concentrations added.

The slopes of the lines in **Fig. S6A** are plotted in **Fig. S6B**, as the amount of niclosamide dissolved out of the tablet material per minute (basically dm/dt). Initial rates over the first 5, 10 and 15 mins were: 14.4μM/min for 1mM; 7.6μM/min for 600μM; and 4.2μM/min for 300μM. Although not known exactly, surface area and mixing were constant for each sample, i.e., the dissolution was carried out using the same ground powder and hence consistent surface area and the same stirring speed of 300rpm for the same stir bar. The initial rate of dissolution increases with increasing amount of niclosamide present, basically reflecting the increased surface area of material available to be dissolved. While only three points, the line does intercept at 0.0 with a linear relationship of 0.014 μM/min per μM of Yomesan-niclosamide equivalent added. Thus, as shown in these and the overall dissolution plots, we can expect that the more niclosamide available the faster it will increase in supernatant concentration. However, as shown next, more powder does not necessarily equate to a greater fraction of niclosamide being extracted, in fact it is quite the opposite.

S6. Niclosamide in Tris Buffered Saline Precipitates or Grows "Wheatsheaf" Crystal Morphologies

As additional context to the needle-shaped morphologies that are formed by growing on the particulate aggregates, it is interesting to compare other systems such as niclosamide precipitation into Tris Buffered Saline Solutions (TBSS). When pure niclosamide is precipitated from an ethanolic solution by solvent exchange into TBSS at pH 8.45, bright red wheatsheaf needle morphologies are rapidly A typical example is shown in **Fig. S7**. Needles (**Fig. S7A**) emanate from what appears to be a nucleating core shown at higher magnification (Fig. S7B). These precipitation experiments for niclosamide, including niclosamide sodium, will form the basis for a subsequent paper [28].

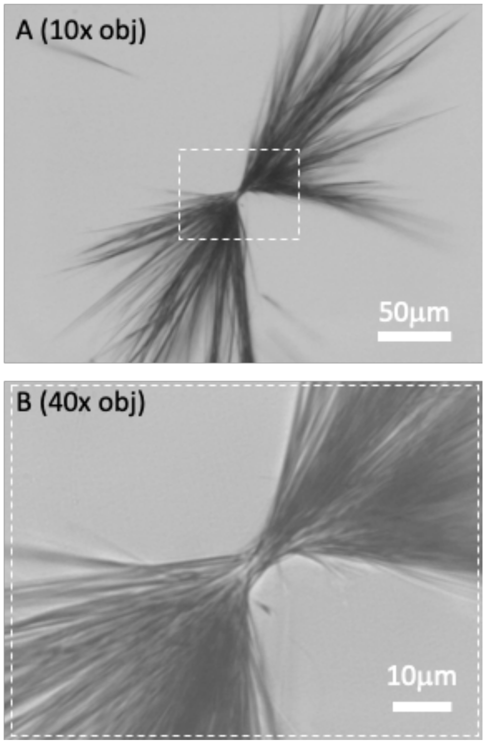

Fig. S7. Wheatsheaf-like morphology (red) of pure niclosamide precipitated at 120µM by solvent exchange into Tris Buffered saline (100mM NaCl) at pH 8.45. **A)** 10x objective with ROI in dotted box; **B)** 40x objective, showing higher resolution image of ROI outlined in **A.**

These data are very preliminary and motivate further pure niclosamide studies with alkali metal salts, where these red crystals are readily formed in the presence of sodium salts and buffers, but only in the higher pH range. The hypothesis to be tested would be that in order to form the sodium salt, there needs to be a considerable amount of the negatively charged niclosamide species (for the Nic^- and Na^+ interaction), and so this occurs best at the higher pH where niclosamide is almost fully deprotonated. The implication from this kind of data of any formulation of niclosamide in aqueous buffer is that, in the presence of alkali metal ions, niclosamide preparations at these slightly elevated pHs will move towards this most stable niclosamide-sodium state. Even here though, at 10µM there is 1,000 times the IC_{100} for preventing viral infection of nasal and bronchial epithelial cells [6].

S7. Comparative UV-Vis Spectra

Because Yomesan contained vanillin, the UV-Vis spectrum of vanillin needed to be subtracted from all subsequent spectra for the Yomesan-niclosamide dissolution. Presented here for completion are the typical baseline-corrected spectra and the 30s-subtracted spectra for the two main Specific Aims: The Concentrations at pH 9.35 and the pH dependence. Only the consecutive spectra where there is increasing concentration are shown.

S7.1 Concentration Dependence

The concentration dependence for the dissolution of niclosamide from Yomesan powder was carried out at pH 9.35 at 300μM, 600μM and 1mM initial added Yomesan-niclosamide equivalent concentrations. Shown in **Fig. S8** are the three series of UV-Vis spectra that generated the supernatant niclosamide concentrations plotted in the main text in **Figs 4**, **5** and **6**. In the left-hand column, are the baseline-corrected spectra, and in the right-hand column are the same data but with the 30s spectrum subtracted from all subsequent time points. The 30s spectrum is shown as a dashed line for comparison and to show the effect of subtracting what is essentially the vanillin spectrum.

300μM Fig. S8A

At 300μM added Yomesan niclosamide-equivalent, absorbance spectra for all 180mins progressively increase in intensity for each time point. There was actually no peak or decrease and as shown in **Fig. 4A** (main text), all points up to 180mins were used to give a logarithmic fit. The absorbances and hence niclosamide concentrations did not max-out the instrument. A shown in the right-hand spectra the 30s spectrum (orange dashed line) were subtracted to give the 333nm values reported as niclosamide concentrations versus time after addition of the Yomesan powder in **Fig.4A**.

600μM Fig. S8B

At 600μM added Yomesan niclosamide-equivalent, baseline corrected absorbance spectra shown in the left progressively increased in intensity until ~ 60 mins when the absorbances started to reach the limit of the instrument. The spectra became noisy, but they average out to values that were similar to the 2x dilution of the same samples. Thus, the limits of the instrument were approached but not exceeded. The 30s spectrum (orange line) at this higher initial Yomesan concentration did increase (doubled compared to the 300μM sample) as also shown in **Fig. 2A** and **B** (main text). Subtracting this 30s spectrum (orange dashed line) gave the series of spectra on the right, that increased in intensity out to 150 mins. These spectra were thus baseline-corrected, 30s-subtracted and 2x-diluted from the 50min time point onwards and provide the niclosamide concentrations for **Fig 4B** (main text).

1mM Fig. S8C

The baseline corrected spectra for the 1mM sample reached the instrument maximum after only 20 mins of dissolution. As shown in **Fig. 4C** (main text) the values plateaued, while the 2x diluted samples continued to show the increasing absorbance. They reached a peak after 90mins and then

the absorbances started to decrease. Thus, only the first 90 mins are shown as increasing absorbance.

Again, the amount of vanillin that came with this higher 1mM Yomesan niclosamide-equivalent concentration, is reflected in the absorbance intensity of the 30s spectrum (orange line) and data on **Fig. 2A** and **B** (main text). Again, subtracting this 30s spectrum gave the baseline corrected, 30s subtracted and 2x diluted spectra form which the data in **Fig. 4C** (main text) was derived.

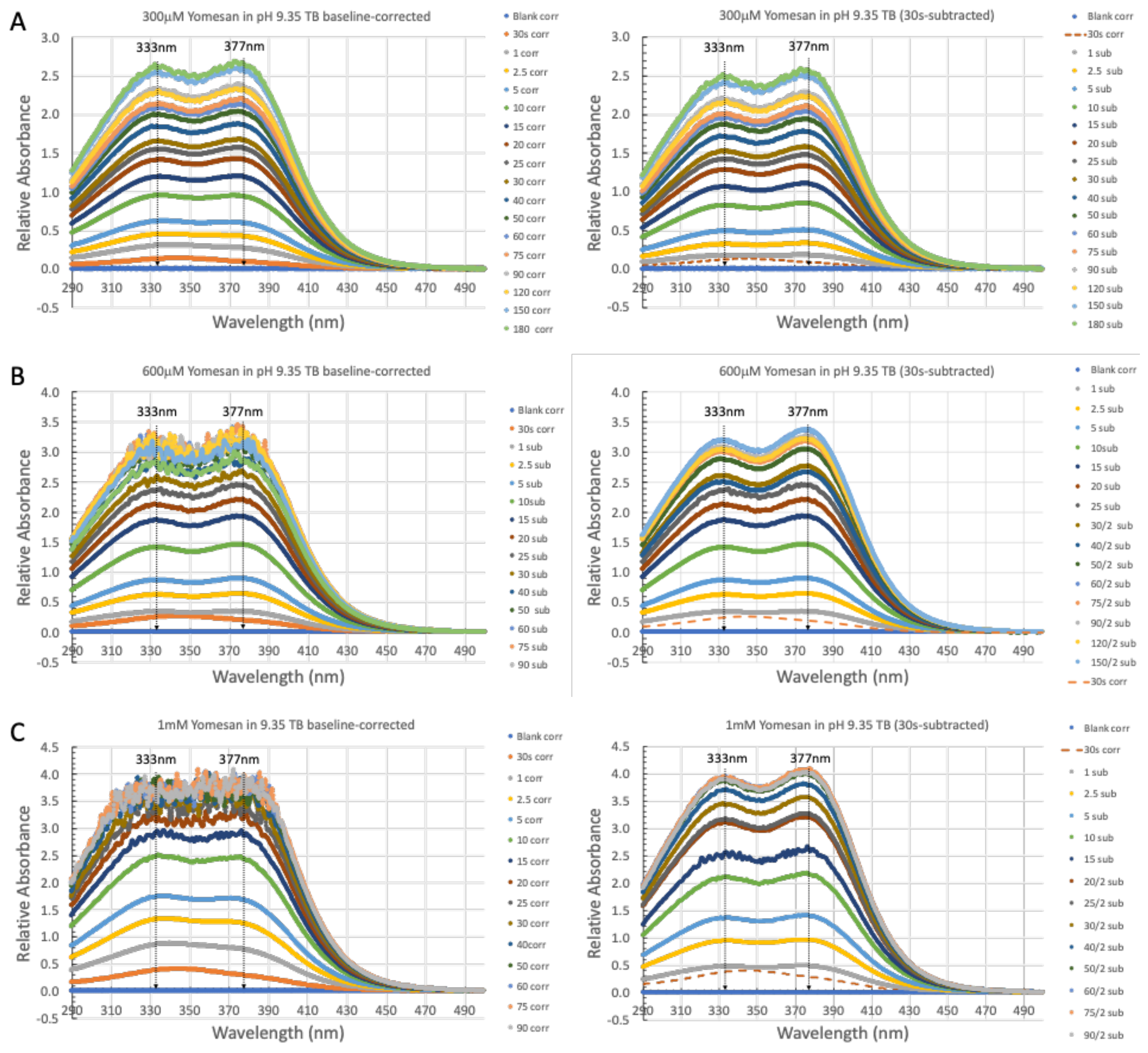

Fig S8. UV-Vis spectra for dissolution of niclosamide at initial added Yomesan-niclosamide-equivalent concentrations of **A)** 300µM, **B)** 600µM and **C)** 1mM in pH 9.35 Tris Buffer. (Left) are the baseline corrected spectra; (right) 30s-subtracted spectra showing the 30s impurity spectrum in dashed orange. For 600µM and 1mM, the spectra that reach the limit of the Spectrometer are diluted 2x then absorbance is doubled. (NOTE: different y-axis scales are used to accommodate the data)

S7.2 pH Dependence

Measuring the pH-dependence of the dissolution and extraction process was the second important part of the study that further optimized the extraction of niclosamide from Yomesan tablet material. The UV-Vis spectra for each nominal Tris buffer pH (7.41, 8.35, 8.85, and 9.35) are shown in **Fig. S9** and correspond to the niclosamide concentrations plotted in **Fig. 9** (main text)

pH 7.41 Fig S9A

The spectra on the left in **Fig S9A** at pH 7.41 for a 300μM Yomesan niclosamide-equivalent concentration were dominated by the vanillin signal. They resemble those in **Fig. S3A** for vanillin at pH 7.displaying the two peaks at 316nm and 347nm. However, subtracting the 30s spectrum successfully revealed the characteristic double-peaked profile of niclosamide as shown in the right-hand spectra in **Fig. S9A**. These spectra gave the niclosamide concentrations plotted in **Fig. 9A** (main text) where the dissolution was logarithmic for the first 50 mins, and achieved a peak supernatant niclosamide of only 4μM. The niclosamide concentration essentially plateaued over the next 2 hrs and this is evident from the overlapping spectra. The dotted orange line for the 30s spectrum shows how much this vanillin "impurity" would have compromised the measurement of supernatant niclosamide concentrations if not subtracted.

pH 8.35 Fig S9B

At pH 8.35, 600μM Yomesan niclosamide equivalent was initially added to the tris buffer. The absorbances peaked at ~50 - 60mins, and so only the first 90 mins are shown, (the slight decreases for the 75mins (orange) and 90 min spectra (grey) are evident in the baseline corrected series on the left of **Fig. S9B**. In these spectra the shape is skewed, but again, subtraction of the 30s spectrum (orange dashed line) reveals the characteristic double-peaked spectra of niclosamide that gave the data in **Fig. 9B** (main text).

pH 8.85 Fig S9C

Similarly, for pH 8.85, the UV-Vis absorbance spectra increase in intensity, and for this pH did not peak until 150 mins, and so all 180 min spectra are shown in **Fig. S9C**. Subtraction of the 30s spectrum (orange dotted line) gave the niclosamide spectra in the right-hand image of **Fig. S9C** and provided the data for the niclosamide concentrations plotted in **Fig 9C** (main text).

pH 9.35 Fig S9B

Finally, the data for the 600μM Yomesan niclosamide equivalent added to pH 9.35 TB in **Fig. S9D**, are the same as above in **Fig. S8B**. Absorbance peaked at ~90 mins and while they did not reach the maximum om the instrument, the 2x-dilution spectra are shown to 150 mins. These spectra provided the data for the plot of niclosamide concentrations in **Figs. 4B** and **9D** (main text).

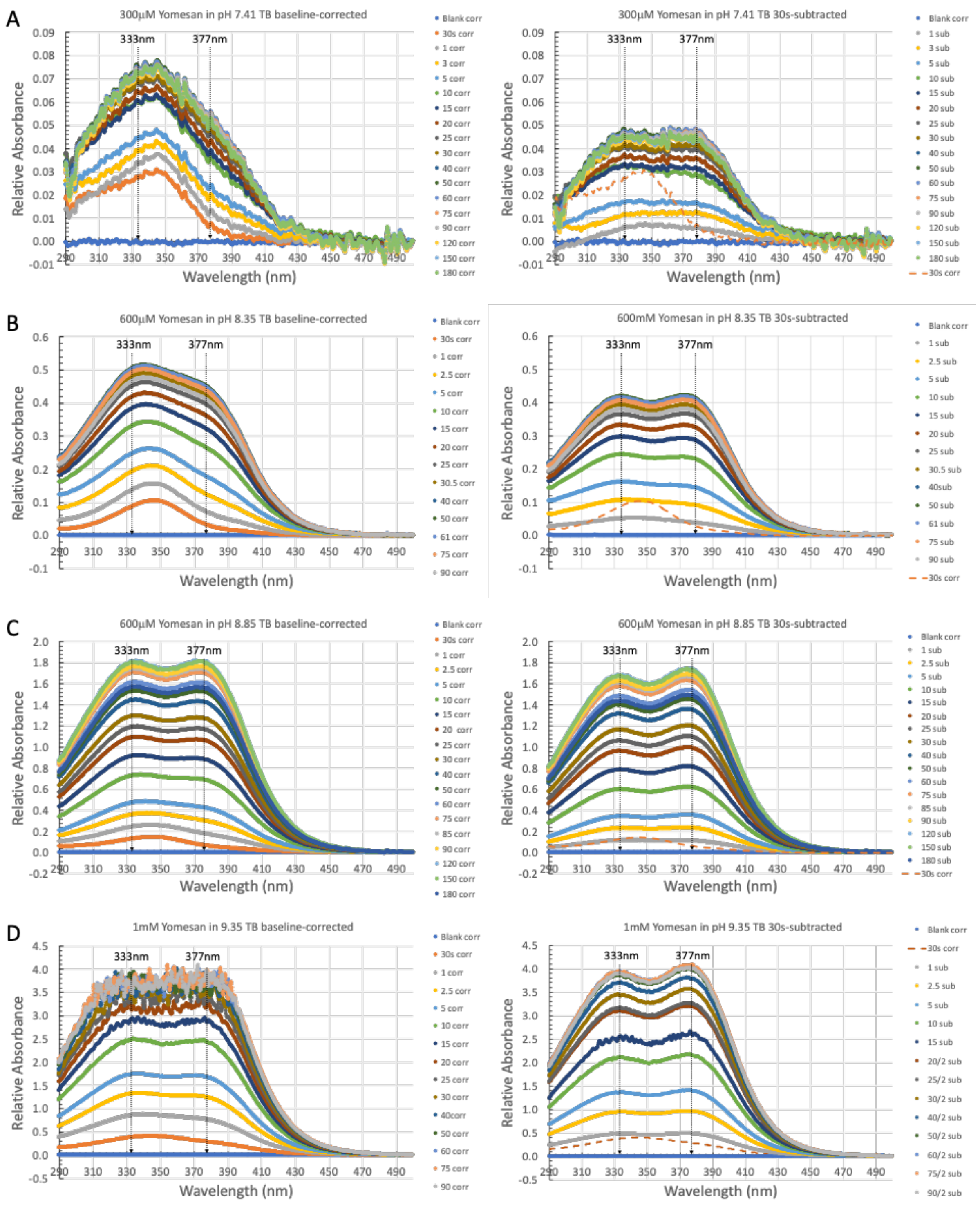

Fig S9. UV-Vis spectra for dissolution of niclosamide as a function of nominal pH. **A)** 300μM in pH 7.41; **B)** 600μM in pH 8.35; **C)** 600μM in pH 8.85; **D)** 600μM in pH 9.35; (Left) are the baseline corrected spectra; (right) 30s-subtracted spectra showing the 30s impurity spectrum in dashed orange. For 600μM and 1mM, the spectra that reach the limit of the Spectrometer are diluted 2x then absorbance is doubled. (NOTE: different y-axis scales are used to accommodate the data).

S8. Comparative Dosing Between Yomesan and an Oral or Nasal Niclosamide Spray

Niclosamide is available in 500mg tablets (Yomesan, Bayer, [18]) as an oral treatment for gut parasites. The typical dosing for GI parasites is 4 x 500mg (2gms) of niclosamide, chewed thoroughly in the mouth or made into a paste in a little water for children. Such dosing is taken orally, and so the idea here was to determine if and to what extent niclosamide could actually be pH-extracted (no organic solvent used) from commercially available and already regulatory-approved tablets. If so, the obtained niclosamide solution would be very close to an already approved dosage composition, and, if used as an oral spray, would essentially be applied locally to the same buccal and throat epithelium as the "thoroughly chewed" 2 grams of Yomesan tablets. A rule-of-thumb is that, for the 327.1g/mol molecular weight niclosamide, a 30μM solution of niclosamide is just 10mg/L.

Because of Niclosamide's low ~1μM – 2μM solubility at neutral pH, it is calculated that, of these 2 grams, only ~10 micrograms would be immediately bioavailable if dissolved in saliva or in a 30mL tap-water paste. Thus, in 30mLs of tap water/saliva solution, a 1μM solution, (30 x 10^{-3}L) x (1 x 10^{-6}M/L) x (327.1g/mol) = 9,813 x 10^{-9}g, ~ **9.8 micrograms** of the niclosamide in Yomesan tablets that would dissolve out in the mouth. For a 300μM niclosamide solution at pH 9.2, a 100μL oral spray of a 300μM Niclosamide solution (100 x 10^{-6}L x 300 x 10^{-6}M/L x 327.1g/M = 327.1 x 10^{-9}) i.e., ~**9.8 micrograms** in the oral spray. And so, at 300μM Niclosamide, the oral sprayed dose is on the same order as what is estimated from the immediately bioavailable oral tablets. A preventative prophylactic nasal spray could use a 15x lower concentration of 20μM (~**0.65 microgram** per nasal spray), which, according to our recent cell studies [9], would be 2,000 times the 10nM concentration needed to stop viral infection of nasal and bronchial cells; and it would be safe in terms of nasal cell viability for frequent use.

References Cited

1. Shu M, Man Y, Ma H, Luan F, Liu H, Gao Y. Determination of Vanillin in Milk Powder by Capillary Electrophoresis Combined with Dispersive Liquid-Liquid Microextraction. Food Analytical Methods. 2016;9(6):1706-12. doi: 10.1007/s12161-015-0347-8.

2. NIST: Vanillin https://webbook.nist.gov/cgi/cbook.cgi?ID=121-33-5. (2022). Accessed 09/30/2022 2022.

3. Needham D. The pH Dependence of Niclosamide Solubility, Dissolution, and Morphology: Motivation for Potentially Universal Mucin-Penetrating Nasal and Throat Sprays for COVID19, its Variants and other Viral Infections. Pharm Res. 2022;39(1):115-41. doi: 10.1007/s11095-021-03112-x.

4. Shakeel F, Haq N, Siddiqui NA. Solubility and thermodynamic function of vanillin in ten different environmentally benign solvents. Food Chem. 2015;180:244-8. doi: 10.1016/j.foodchem.2015.01.102.

5. Jain P, He, Y., , Yalkowsky SH. Handbook of Aqueous Solubility Data. 2nd ed. CRC Press.; 2010.

6. Kelleher Z, Needham D, Barkauskas C. Niclosamide Stops SARS-CoV2 Virus Replication in Human Nasal and Bronchial Epithelial Cells at 10nM Concentration --100x less than the IC100 Previously Reported in the More Robust Vero6 Cells (in preparation). 2022.
